# Yeast Plasma Membrane Fungal Oligopeptide Transporters Display Distinct Substrate Preferences despite Their High Sequence Identity

**DOI:** 10.3390/jof7110963

**Published:** 2021-11-12

**Authors:** Carmen Becerra-Rodríguez, Géraldine Taghouti, Perrine Portier, Sylvie Dequin, Margarida Casal, Sandra Paiva, Virginie Galeote

**Affiliations:** 1 SPO, Univ. Montpellier, INRAE, Institut Agro, F-34060 Montpellier, France; carmen.becerra-rodriguez@inrae.fr (C.B.-R.); sylvie.dequin@inrae.fr (S.D.); 2 Centre of Environmental and Molecular Biology, Department of Biology, Campus of Gualtar, University of Minho, 4710-057 Braga, Portugal; mcasal@bio.uminho.pt (M.C.); spaiva@bio.uminho.pt (S.P.); 3 Univ. Angers, Institut Agro, INRAE, IRHS, SFR QUASAV, CIRM-CFBP, F-49000 Angers, France; geraldine.taghouti@inrae.fr (G.T.); perrine.portier@inrae.fr (P.P.)

**Keywords:** Fungal Oligopeptide Transporters (Fot), oligopeptide transport, *Saccharomyces cerevisiae*, phenotype microarrays, GFP labeling

## Abstract

Fungal Oligopeptide Transporters (Fot) Fot1, Fot2 and Fot3 have been found in *Saccharomyces cerevisiae* wine strains, but not in strains from other environments. In the *S. cerevisiae* wine strain EC1118, Fot1 and Fot2 are responsible for a broader range of oligopeptide utilization in comparison with strains not containing any Fot. This leads to better fermentation efficiency and an increased production of desirable organoleptic compounds in wine. Despite the benefits associated with Fot activity in *S. cerevisiae* within the wine environment, little is known about this family of transporters in yeast. The presence of Fot1, Fot2 and Fot3 in *S. cerevisiae* wine strains is due to horizontal gene transfer from the yeast *Torulaspora microellipsoides*, which harbors Fot2Tm, FotX and FotY proteins. Sequence analyses revealed that Fot family members have a high sequence identity in these yeast species. In this work, we aimed to further characterize the different Fot family members in terms of subcellular localization, gene expression in enological fermentation and substrate specificity. Using CRISPR/Cas9, we constructed *S. cerevisiae* wine strains containing each different Fot as the sole oligopeptide transporter to analyze their oligopeptide preferences by phenotype microarrays. The results of oligopeptide consumption show that Fot counterparts have different di-/tripeptide specificities, suggesting that punctual sequence divergence between *FOT* genes can be crucial for substrate recognition, binding and transport activity. *FOT* gene expression levels in different *S. cerevisiae* wine strains during enological fermentation, together with predicted binding motifs for transcriptional regulators in nitrogen metabolism, indicate that these transporters may be under the control of the Nitrogen Catabolite Repression (NCR) system. Finally, we demonstrated that Fot1 is located in the yeast plasma membrane. This work contributes to a better understanding of this family of oligopeptide transporters, which have demonstrated a key role in the utilization of oligopeptides by *S. cerevisiae* in enological fermentation.

## 1. Introduction

Oligopeptides, often simply referred to as peptides, are short chains of two to nine amino acid residues linked by an amide type bond mainly present in nature as a result of proteolytic processes. They constitute a highly diverse source of nitrogen and carbon for microorganisms. As for other nutrient molecules, the ability to consume oligopeptides from the environment can represent a crucial competitive advantage in a microbial ecosystem. In the eukaryotic model organism *Saccharomyces cerevisiae*, three systems for oligopeptide transport are well documented: Ptr2, a Proton-dependent Oligopeptide Transporter (Pot/Ptr, Transporter Classification (TC) number: 2.A.17) [1] is the best-known di- and tripeptide transporter in yeast, together with the allantoate and ureidosuccinate permease Dal5 (TC 2.A.1.14.4), which also displays dipeptide transport activity [2]. Two members of the Oligopeptide Transport (Opt) family (TC 2.A.67), glutathione transporter Opt1 and its paralogue Opt2, can transport tetrapeptides and also pentapeptides in the specific case of Opt1 [3,4]. Finally, new oligopeptide transporters from eukaryotes have been identified from a soil sample. Phylogenetic analyses indicated that these transporter sequences only grouped with other fungal species and were not homologous to either Ptr2, Dal5 or Opt members; as a result, a novel family termed Fungal Oligopeptide Transporters (Fot) was defined and characterized as proton-driven membrane transporters in fungi able to import di-and tripeptides [5]. Fot proteins were related to fungal members of the Amino Acid/Auxin Permease family (TC 2.A.18.4.) [5]. Interestingly, *S. cerevisiae FOT* genes are only present in wine strains, and not in strains from other environments. The genes *FOT1*, *FOT2* and *FOT3*, found in different *S. cerevisiae* wine strains, are the result of a horizontal gene transfer and subsequent gene conversions between *FOTX* and *FOT2Tm* from the yeast *Torulaspora microellipsoides*, which also contains *FOTY* [6,7]. Fot family members display a high sequence identity at both the protein and gene level, from 90% identity between *FOTY* and *FOT2* genes to 98% between *FOTX* and *FOT3* [7].

The deletion of *FOT1* and *FOT2* genes in the *S. cerevisiae* 59A strain, a haploid derivative of commercial wine strain Lalvin EC1118^®^, led to a 35% drop in oligopeptide-derived nitrogen consumption. Compared to the deletion mutant, wild-type strain 59A had higher biomass accumulation by the end of fermentation, which is consistent with a higher nitrogen consumption [7]. Strain 59A also showed two remarkable features as a consequence of the higher consumption of glutamate/glutamine-rich oligopeptides due to Fot1 and Fot2 activity: first, a higher cell viability by the end of fermentation in comparison to fot1fot2Δ [7], and secondly, a more positive wine organoleptic balance due to a lower production of acetate and higher levels of ester acetates and fusel alcohols [8]. This effect on the production of fermentative organoleptic compounds was later demonstrated to be dependent on the source of peptides present in the must [9]. With higher biomass and viability as evident fitness indicators, these results suggest that Fot acquisition by *S. cerevisiae* wine strains confers a competitive advantage in the wine environment [7,8,10].

Despite the important role that Fot family members play in the adaptation of *S. cerevisiae* to the wine fermentation environment, little is known still about this family of transporters. The most evident question that arises is the biological significance of the five different Fot members currently known, considering their high sequence identity. To answer this question, we followed a CRISPR/Cas9 strategy to construct *S. cerevisiae* wine strains containing single Fot members as the sole oligopeptide transporter. Analysis of oligopeptide preferences by these strains revealed that Fot1, Fot2, Fot3, FotX and FotY have distinct substrate specificities, highlighting the importance of the amino acid nature and position within the oligopeptide for their consumption as substrates. Moreover, we provide evidence that *FOT* expression is dependent on the strain, stage of enological fermentation and composition of the yeast assimilable nitrogen. Additionally, using fluorescence microscopy and co-localization studies with GFP labeling, we demonstrated that Fot1 is localized in the yeast plasma membrane. With this work we aimed at advancing the knowledge regarding the expression and function of Fot family members, which have a key role in the adaptation of *S. cerevisiae* to wine environments.

## 2. Materials and Methods

### 2.1. Yeast Strains and Fermentation Conditions

In this study, we worked with *S. cerevisiae* strains 59A and MTF2533, haploid derivatives of the commercial wine strains Lalvin EC1118^®^ and LMD1, respectively [11,12]. We used a 59A version in which gene *AMN1* has been deleted to avoid cell aggregation [7,13,14]. Fermentations were carried out in 1.2 L glass fermenters inoculated with 10^6^ cells/mL. We used natural Colombard grape must (Caussens, France, 2019) containing 183 g/L of sugars and 257 mg/L of yeast assimilable nitrogen (ammonium and amino acids). To mimic the conditions and composition of the natural must, a synthetic must (SM) was prepared with 183 g/L glucose/fructose, 257 mg/L yeast assimilable nitrogen (ammonium and free amino acids), 1.12 mg/L oleic acid and 3.75 mg/L ergosterol, at pH 3.3 [15]. Cells were grown on double overnight pre-cultures, first on YPD (1% yeast extract, 2% bactopeptone, 2% glucose) at 28 °C in 10 mL flasks with shaking (180 rpm) and then on SM in the same conditions before inoculation of fermenters. Strains with antibiotic resistance were selected on YPD medium supplemented with 300 µg/mL hygromycin B (Sigma, 31282–04–9, Saint Louis, MO, USA), 200 µg/mL G418 (Sigma A-1720) and/or 100 µg/mL nourseothricin (Werner, 96736–11–7, Meisenweg, Germany).

### 2.2. Strain and Plasmid Construction

Tandem genes *FOT1–FOT2* were replaced by a *KANMX4* cassette in strain 59A, obtaining the strain fot1fot2Δ. The genes of the non-Fot oligopeptide transporters in 59A, i.e., *OPT1*, *OPT2* and *DAL5*, were then deleted from 59A and fot1fot2Δ using the CRISPR/Cas9 system with two plasmids and one repair fragment [16]. This system requires first a transformation with plasmid pCfB2513 for the expression of Cas9; in a second transformation, a repair fragment and plasmid pMEL15 [17] containing the guide RNA (gRNA) cassette for the target gene are introduced. Genes *DAL5*, *OPT1* and *OPT2* were sequentially deleted, with the consequent pMEL15 loss in between to ensure the correct selection of transformants. pMEL15 vectors containing the gRNA cassettes were generated by PCR, whereas the repair fragments consisting of disrupted versions of the target genes were designed and amplified from a pEX-A128 plasmid (Eurofins Genomics, Ebersberg, Germany). *PTR2*, which is the gene coding the best-known dipeptide transporter in *S. cerevisiae*, is not functional in 59A, and its deletion was therefore not required [5]. Deletion of non-*FOT* oligopeptide transporter genes in fot1fot2Δ resulted in the strain *opt1Δ opt2Δ dal5Δ fot1fot2Δ::KANMX4*, termed PepKO, which was a knockout strain for oligopeptide transport and constituted a platform strain for the insertion of single *FOT* genes. Using CRISPR/Cas9, each *FOT* gene was inserted in a substitution of the *KANMX4* cassette in PepKO. In this way, all *FOT* genes were individually located in the original *FOT1–FOT2* locus and therefore were under the regulation of the *FOT2* promoter and *FOT1* terminator. All these transformations were carried out using the lithium acetate method for yeast transformation [18]. The different strains containing single *FOT* genes were confirmed by sequencing.

Plasmids containing the *GFP* gene fused to *FOT1* at its 5′ or 3′ ends (pGFP–Fot1 and pFot1–GFP, respectively) were constructed by Gibson assembly (New England Biolabs, Ipswich, MA, USA) and confirmed by digestion with restriction enzymes. The strains used and constructed in this study are listed in Table 1; primers and plasmids used in this study are listed in Tables S1 and S2, respectively.

### 2.3. Phenotype Microarray Assays for Di- and Tripeptide Consumption

The di/tripeptide utilization profile of the different strains was performed using the Biolog (Hayward, CA, USA) Phenotype MicroArrays (PM) system combined with the OmniLog reader (Biolog, Hayward, CA, USA) at the COMIC facility of SFR Quasay (University of Angers, France). This technique enables the monitoring of the consumption of a range of substrates over time through a colorimetric method. We used four PM plates (PM3B for nitrogen sources, PM6, PM7 and PM8 for peptide nitrogen sources) containing a total range of 270 dipeptides and 14 tripeptides, with a negative control and L-glutamine as a positive control per plate. Cultures were incubated on yeast nitrogen base plates without amino acids (YNB, Difco BD 91940, 6.7 g/L; 2% glucose, 2% agar) at 28 °C in duplicate and prepared for incubation on PM plates as in [19]. PM assays were performed at 30 °C over 72 h. After this time, data from the Omnilog system were retrieved using Kinetics software v1.30. Area under the curve was calculated and assigned as consumption values. We considered a strain to have utilized a di/tripeptide when the consumption value after 72 h on this nitrogen source represented more than 20% of L-glutamine consumption; this threshold was taken as the positive control and therefore the glutamine consumption value for each strain in each plate represented 100% consumption. Below 20%, the growth signal would possibly be mistaken with spontaneous reduction of the tetrazolium dye or reduction by the remaining yeast inoculum [5]. Levels of consumption from 0 to 5 were established based on the consumption value of L-glutamine, the positive control in each plate. Level 0, 0–20% of consumption on L-Gln; 1, 21–40%; 2, 41–60%; 3, 61–80%; 4, 81–100%; 5, >100%.

### 2.4. Gene Expression Analysis

Gene expression was analyzed in three independent cultures for each medium and yeast strain. Cells were sampled at 10% (growth phase) and 40% (stationary phase) of the total CO_2_ produced from each must during fermentation (see Figure S3). A total of 10^9^ cells were collected for each strain/must/fermentation stage, washed with diethylpyrocarbonate (DEPC)-treated water, frozen in methanol and stored at −80 °C for RNA extraction. RNA was isolated using Trizol reagent (Gibco BRL, Life Technologies, Waltham, MA, USA) and purified by isopropanol precipitation using a RNeasy kit (Qiagen, Hilden, Germany). RNA samples were retro-transcribed into cDNA and used for Quantitative Polymerase Chain Reaction (qPCR). Following the recommendations of the Real-Time PCR system manufacturer (Applied Biosystems, Waltham, MA, USA), *FOT* gene expression was quantified by a relative standard curve method using genomic DNA of the yeast strain. Expression values were subsequently normalized with those of the house-keeping gene *SCR1* (Small Cytoplasmatic RNA 1), which is commonly used as reference gene in *S. cerevisiae* due to its high expression stability. Primers were designed for specific amplification of *FOT1*, *FOT2*, *FOT3* and *SCR1* (Table S1).

### 2.5. Epifluorescence and Confocal Microscopy

N-(3-Triethylammoniumpropyl)-4-(6-(4-(Diethylamino) Phenyl) Hexatrienyl) Pyridinium Dibromide or FM4–64 dye (Invitrogen, Waltham, MA, USA) was used as a fluorescence differential marker for plasma membrane (excitation 558 nm, emission 734 nm). A total of 100 µL of cells was collected at the exponential phase and incubated with 80 µM FM4–64 at 4 °C with agitation (1500 rpm). After a 1-h incubation, cells were spun at 700 g for 3 min at 4 °C and prepared for immediate visualization (adapted from [20]). Cells were visualized with a 100×/1.3 oil objective under an Axio Imager Cam MRM A2 microscope (Carl Zeiss, White Plains, NY, USA) equipped with an excitation source and a range of filters. For this study, we used filter 38 (excitation BP 470/40 and emission BP 525/50) for visualizing GFP (488 nm excitation and 530 nm emission) and filter 20 (excitation: BP 546/12, emission: BP 575–640) for visualizing FM4–64 staining. Images of epifluorescence microscopy were captured and processed with ZEN 2012 software, version 1.1.2.0 (blue edition; Carl Zeiss, New York, NY, USA). Confocal microscopy was performed with a Confocal Leica 8 (Leica Microsystems CMS, Germany) with a 40×/1.1 oil objective. Fluorescence emission was collected at 500–540 nm for GFP and at 600–650 nm for FM4–64 by sequential acquisition. Images from confocal microscopy were treated and analyzed with LAS X (Leica) and Fiji [21] software.

### 2.6. Analysis of Promoter Regions

Characterization of promoter regions was carried out using the YeTFaSCo database v1.02 [22] to identify binding sites for transcription factors. A 500 bp region upstream the start codon was analyzed against the expert-curated, non-dubious set of transcriptional factors. Results were filtered for a value of maximum score higher than 95% identity.

### 2.7. Data Treatment and Statistical Analysis

Data were treated and analyzed using R v4.1.0 (R Core Team 2021) and RStudio (RStudio Team 2020). Dedicated packages were used depending on the purpose: tidyverse [23] for data manipulation and visualization; heatmaply [24] for heatmap construction and agricolae package (Mendiburu and Yaseen, 2020) for statistical analysis.

## 3. Results

### 3.1. Evaluation of Substrate Specificity in Fot Family Members by Phenotype Microarrays

**Figure 1:**
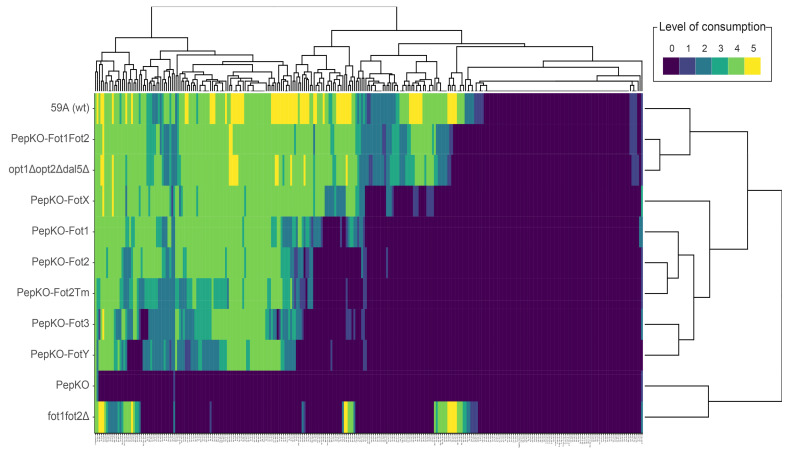
Fot have different peptide specificities. The heatmap represents the consumption of di- and tripeptides (columns) by Fot-containing strains (rows). Strains are sorted by consumption preferences similarity, represented by the cladogram to the right. The cladogram on top of the heatmap ranks the di- and tripeptides according to their preferential consumption.

We sought to characterize the substrate preference of each Fot by phenotype microarrays. For this purpose, we first generated a complete knockout strain for oligopeptide transport by deleting *OPT1*, *OPT2* and *DAL5* with CRISPR/Cas9 and substituting *FOT1–FOT2* tandem genes by a *KANMX4* cassette (Table 1). This knockout strain, denominated PepKO, was unable to consume any of the 284 oligopeptides in the microarray, except for a weak consumption of His–Pro, Thr–Ser and γ-Glu–Gly dipeptides (Figure 1, second to last row in the heatmap; Table S3). The consumption of γ-Glu–Gly was probably due to the activity of the general amino acid transporter Gap1, which has also been reported to transport γ-glutamyl dipeptides [25]. In comparison, the wild-type strain 59A consumed 195 dipeptides and 12 tripeptides from a total range of 270 dipeptides and 14 tripeptides (Figure 1, first row in the heatmap). This result confirms that we had deleted all significant di- and tripeptide transporters in strain 59A. Using CRISPR/Cas9, each *FOT* gene was inserted into the PepKO strain, substituting the *KANMX4* cassette. In this way, we generated *S. cerevisiae* strains containing single Fot family members as sole oligopeptide transporters (Table 1), with *FOT* genes individually located in the original *FOT1–FOT2* locus and therefore were under the regulation of the *FOT2* promoter and *FOT1* terminator. This allowed the characterization of independent transporters that were under the same gene regulation.

Strains containing single Fot members showed different peptide specificities. In the cladogram to the right in Figure 1, 59A is grouped with strains expressing Fot1 and Fot2 but not the non-Fot oligopeptide transporters, which are opt1Δopt2Δdal5Δ and PepKO–Fot1Fot2. Strain opt1Δopt2Δdal5Δ resulted from *DAL5*, *OPT1* and *OPT2* deletion in the wild-type strain 59A, while PepKO–Fot1Fot2 originates from the re-insertion of *FOT1–FOT2* in PepKO. Both strains consumed, respectively, 189 and 191 oligopeptides from a total of 207 oligopeptides consumed by the wild-type atrain, only differing in Gly–Leu and Lys–Trp, which were weakly consumed (consumption level = 1) by PepKO–Fot1Fot2 (Table S3). This result confirms that the genome insertion of *FOT* genes on the platform strain PepKO did not perturb the oligopeptide consumption phenotype. Strain fot1fot2Δ was able to consume 12 dipeptides and 2 tripeptides that were also consumed by the wild-type and, conversely, not consumed by opt1Δopt2Δdal5Δ or PepKO–Fot1Fot2. Therefore, this 14-oligopeptide fraction, characterized by the presence of glycine at the oligopeptide N-terminus, is specifically consumed by strains expressing the non-Fot oligopeptide transporters, Dal5, Opt1 or Opt2. Consequently, these results confirm that Fot1 and Fot2 are the main di- and tripeptide transporters in the wine strain 59A, as we previously reported under different experimental conditions [7].

Among the strains containing single Fot, the strain with the widest range of oligopeptide utilization was the one expressing FotX, consuming 152 di- and tripeptides (Figure 1, forthth row in the heatmap). Consumption profiles for cells expressing Fot2Tm and Fot2 from *S. cerevisiae* were highly similar (Figure 1, sixth–seventh rows in the heatmap), an expected result considering they share the same protein sequence. Fot1 peptide preferences were closer to those of Fot2 than to the ones of FotX or Fot3, which was an interesting result considering that Fot1 shares 96.21% protein sequence identity with Fot2 and, respectively, 98.11% and 97.26% with FotX and Fot3 (Figure S1) ([7]; reviewed in [10]). Fot3 and FotY were sub-grouped together in the dendrogram (Figure 1) since they had a comparatively shorter range of oligopeptide utilization, i.e., 110 and 99 out of 284 di- and tripeptides, respectively. The fact that the Fot3 strain had a peptide utilization closer to the FotY strain than to the Fot1 or FotX strains was a priori unexpected, considering that Fot3 displays 97.26% and 98.8% amino acid sequence identity, respectively, with these transporters, but only 92.21% with FotY. This result may indicate that the localized sequence differences between Fot3 and FotX can result in a dramatic change in transport capabilities.

It was remarkable that Fot1 and Fot2 as sole transporters were not able to match the transport specificity of strains containing both transporters, PepKO–Fot1Fot2 and opt1Δopt2Δdal5Δ. Strains expressing Fot1–Fot2 consumed 59 oligopeptides more than strains with Fot1 (PepKO–Fot1) or Fot2 (PepKO–Fot2) only (Figure S2A). This difference did not simply come from an expected additive effect of Fot1 and Fot2 acting together, since the level of consumption of 21 out of these 59 oligopeptides was 4 in strains harboring Fot1–Fot2 (high consumption) versus level 0 in strains with Fot1 or Fot2 (Figure S2B). The group of 59 oligopeptides transported by Fot1 and Fot2 together but not singly were rich in Gly and, to a lesser extent, in Trp, Glu, Pro and Asp, with the three latter particularly abundant at the C-terminal position (Figure S2C). This result suggests a possible interaction between Fot1 and Fot2 that causes a modification in their transport abilities and specificities.

### 3.2. Specificity of Fot Members Depends on the Type of Amino Acid Located in the Oligopeptide N-Terminus

**Figure 2:**
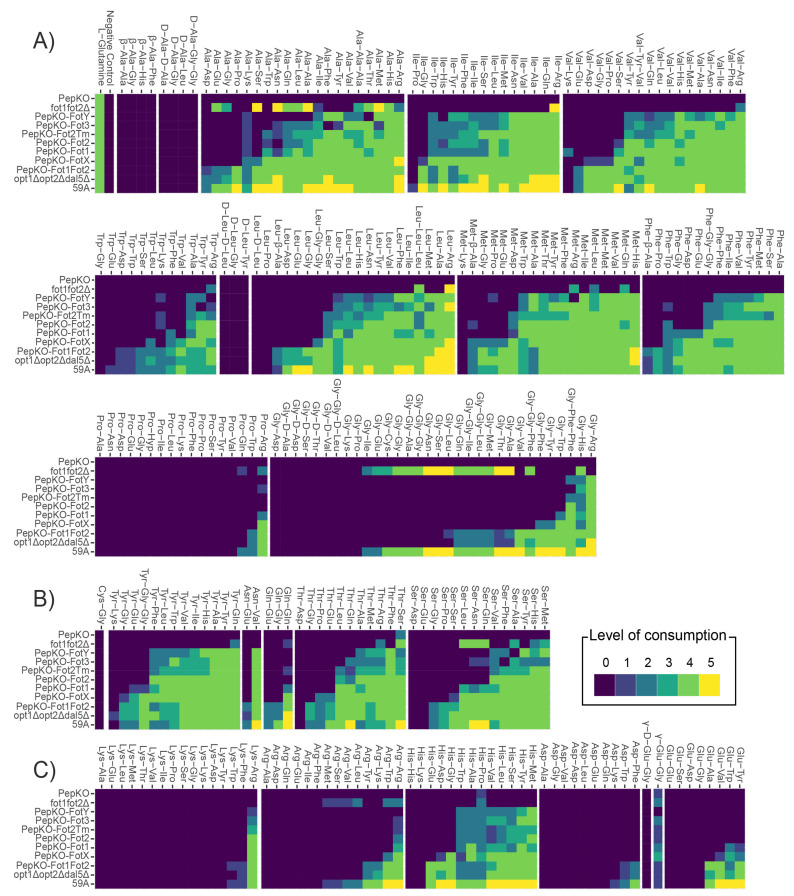
Oligopeptide preferences depend on the character of the amino acid residues. In this figure, di- and tripeptides are classified based on the amino acid type at the N-terminal position. Levels of consumption are expressed in comparison to the consumption value of L-glutamine, as a positive control in each plate; level 0, 0–20% of consumption on L-Gln; level 1, 21–40%; level 2, 40–60%; level 3, 60–80%; level 4, 80–100%; level 5, >100%. (**A**) Peptides with hydrophobic amino acids. (**B**) Peptides with polar, uncharged amino acids; (**C**) Peptides with charged amino acids.

The results of the di- and tripeptide consumption from the Phenotype Microarrays (PM) plates were categorized based on the type of amino acid located at the N-terminal position (Figure 2). In this way, we observed how, in general, Fot preferably transport peptides with hydrophobic amino acids at the N-terminus (Figure 2A), with the general exception of Gly-X and Pro-X oligopeptides. Within the group of oligopeptides with hydrophobic amino acids at the N-terminus, those containing Glu, Asp, Lys, Gly or Pro at the C-terminus were weakly or not consumed by Fot-expressing strains, with the exceptions of Phe–Glu, Phe–Asp or Phe–Gly, moderately consumed in the presence of FotX. Peptides with Trp at the N-terminus were less consumed, although Trp-Tyr was highly consumed by strains harboring FotX, Fot1 and Fot2, but moderately so by strains expressing the other transporters. Except for Cys–Gly, peptides with polar, uncharged amino acids in the N-terminus were also generally consumed by strains harboring Fot members (Figure 2B), particularly if these oligopeptides contained Tyr and/or a hydrophobic amino acid at the C-terminus. Peptides containing charged amino acids were generally not consumed by strains with single Fot, either in N- (Figure 2C) or C-terminal positions, with the exception of His-containing peptides, which were moderately to highly consumed when any Fot was present. A general exception to all these observations regarded peptides containing an Arg in the C-terminal position, which were generally well consumed by all strains expressing any single Fot member, while oligopeptides with Arg in the N-terminus were not consumed by strains containing single Fot or Fot1–Fot2 (except for Arg–Trp, Arg–Lys and Arg–Tyr, consumed in a level range of 2 to 4 by opt1Δopt2Δdal5Δ and PepKO–Fot1Fot2; Table S4). Exceptions to this observation were Pro–Arg, highly consumed by FotX and Fot1–Fot2 strains only, and transporter FotY, which did not allow any Met–Arg, Trp–Arg or Pro–Arg consumption. The case of Arg suggests that localization of amino acids within the oligopeptide is also important for oligopeptide recognition as a substrate. Gln was also more frequent at the C-terminal position of peptides consumed by single Fot-containing strains rather than at the N-terminus (Table S4). Moreover, peptides with D-, β- or γ-amino acids were not consumed by any strain, with the exception of γ-Glu-Gly which was weakly consumed by all strains including PepKO.

### 3.3. FOT Genes Expression Depends on the S. cerevisiae Strain Background, Type of Nitrogen Source and Stage of Enological Fermentation

Two *S. cerevisiae* wine strains were selected to analyze the expression of *FOT1*, *FOT2* and *FOT3*. Strain 59A contains *FOT1* and *FOT2* tandem genes, while strain MTF2533 only has *FOT3*. Two conditions of enological fermentation were evaluated: on one hand, a natural Colombard grape must (Caussens, France, 2019) containing 257 mg/L of assimilable nitrogen and 183 g/L of sugars; on the other hand, a synthetic must with the same concentrations of assimilable nitrogen and sugars. Additionally, *FOT* expression was analyzed at two key points of enological fermentation (Figure S3): 10% of fermentation, point at which cells were at mid-log phase of growth and had not yet reached the maximum fermentation rate or *V*_max_, and 40% of fermentation, which corresponded to a time point of fermentation after *V*_max_, when cells were in stationary phase. *FOT* expression was quantified by qPCR and normalized with expression values of the housekeeping gene *SCR1*.

**Figure 3:**
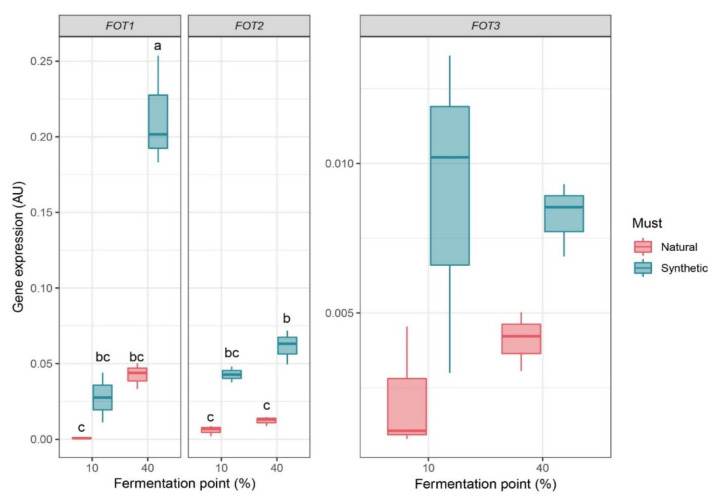
Expression of *FOT* genes at two points of fermentation in different enological conditions. Scale of *Y*-axis for gene expression of *FOT1* and *FOT2* in strain 59A and *FOT3* in MTF2533 differs to facilitate visualization. Letters indicate the statistical groups from a Tukey analysis, *p*-value < 0.001; *n* = 3.

Relative expression values of *FOT* genes were low in both strains (Figure 3), although particularly low for *FOT3* in strain MTF2533. Generally, these expression values were even lower in natural must than in synthetic must, although only statistically significant for *FOT1* and *FOT2* at 40% fermentation. Contrary to this result, we expected a higher expression of *FOT* genes in natural must since grape juice contains oligopeptides as nitrogen source, while synthetic must does not. In addition, *FOT1* and *FOT2* showed higher expression values at 40% of fermentation in both synthetic and natural must; however, the most notable expression was observed for *FOT1* at 40% of fermentation in synthetic must, with a 10-fold expression compared to that at 10%; *FOT1* was also 3.46-fold more expressed than *FOT2* in the same medium and at the same stage of fermentation. Finding higher expression values at 40% of fermentation, i.e., during stationary phase (Figure S3) was also unexpected, since nitrogen is no longer consumed by yeast at this point of fermentation [15]. To predict how *FOT* expression is regulated, we analyzed the promoter regions of *FOT1*, *FOT2* and *FOT3*, and those of *FOTX*, *FOTY* and *FOT2Tm* from *T. microellipsoides* using the scanner tool of the YeTFaSCo database (Table S5). Several binding motifs for transcriptional factors involved in the Nitrogen Catabolite Repression (NCR) such as Gln3 or Cup9 were found in the promoter regions of all *FOT* genes. Gln3 is a transcriptional activator of genes under the regulation of the NCR system, and Cup9 is a transcriptional repressor of both the dipeptide transporter gene *PTR2* and tetrapeptide transporter gene *OPT2* in *S. cerevisiae* [26,27]. Therefore, these findings suggest that *FOT* genes may be under NCR system repression in the presence of preferred nitrogen sources such as ammonium and certain amino acids, which is the case during the growth phase, and be are only expressed when these sources are scarce, which would agree with *FOT1* overexpression at the stationary phase.

### 3.4. Fot1 Is Located in S. cerevisiae Plasma Membrane

To assess the subcellular localization of Fot in yeast, we generated several constructs harboring *FOT1* fused with *GFP* either at its 5′- or 3′ ends, using pRS316 and YEp352 as core plasmids (Table S2), which have distinct origins of replication. The resulting proteins, when expressed in *S. cerevisiae* strain 59A, contained GFP fused either at the N- (pGFP–Fot1) or at the C-terminus (pFot1–GFP) of Fot1. In a preliminary analysis, we found no differences in the expression and localization of the fusion proteins resulting from the use of the two plasmids (data not shown); therefore, we selected the pRS316-based clones for further analysis, termed pFot1–GFP and pGFP–Fot1.

**Figure 4:**
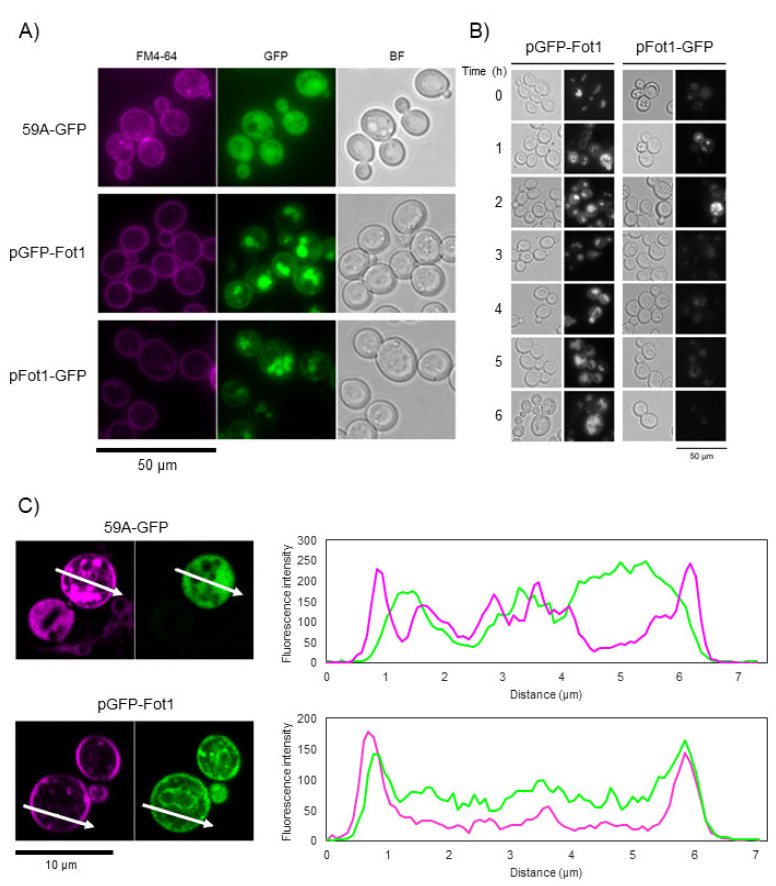
Detection of pFot1–GFP and pGFP–Fot1 localization at the plasma membrane in *S. cerevisiae* 59A cell by fluorescence microscopy. (**A**) Visualization by epifluorescence microscopy of 59A cells expressing pGFP–Fot1 or pFot1–GFP after 1 h of growth in YPD. Strain 59A-GFP was used as control of the subcellular localization; 59A–GFP contains the *GFP* gene in substitution of the *AMN1* gene and it is therefore expressed in the cytosol. FM4–64 dye was used for plasma membrane staining; BF: bright field. (**B**) Visualization by epifluorescence microscopy of 59A cells expressing pFot1–GFP and pGFP–Fot1 over time in growth on YPD. (**C**) Confocal microscopy analysis of GFP and FM4–64 signal co-localization in 59A-GFP strain and 59A containing the pGFP–Fot1 plasmid. FM4–64 and GFP signals are labeled in magenta and green, respectively.

In strain 59A expressing pFot1–GFP or pGFP–Fot1, we observed the Fot1 fusion protein localized at the plasma membrane after 1 h of growth in YPD (Figure 4A). This conclusion was corroborated by FM4–64 staining, a membrane-selective fluorescent dye (Figure S4). We also observed some internal vacuolar fluorescence, most probably due to the overexpression induced by the *TEF* promoter of the plasmid. By contrast, GFP fluorescence signal in control strain 59A-GFP–with the *GFP* gene in substitution of the coding sequence for cytosolic protein Amn1–was observed at the cytosol. Although the GFP signal was detected at the plasma membrane when Fot1 was tagged at the N- or C- terminus, (Figure 4A,B, 1 h of growth in YPD), the signal was clearer and more stable in cells expressing Fot1 with an N-terminal GFP fusion, with GFP fluorescence detected at the plasma membrane even after 6 h (Figure 4B). However, no pFot1–GFP fluorescence was detected at the plasma membrane time after 1 h of growth on YPD. This instability of the pFot1–GFP signal hindered its co-localization study with FM4–64 by confocal microscopy (Figure 4C). Confocal microscopy analysis showed that the GFP signal in control strain 59A-GFP was found in the cytosol and did not overlap with FM4–64 signal peaks at the plasma membrane, despite some partial internalization of the dye into the vacuolar membrane and lumen Fot1 (Pearson’s coefficient = 0.532). By contrast, the peaks of the GFP signal overlapped with those of FM4–64 signal in pGFP–Fot1 (Pearson’s coefficient = 0.773), confirming the Fot1 plasma membrane localization in *S. cerevisiae*.

## 4. Discussion

In the present study, using phenotype microarrays, we confirmed that Fot1 and Fot2 are the main di- and tripeptide transporters in the *S. cerevisiae* strain 59A. Our work sheds new light on a previous study performed during enological fermentation [7]. Individually, Fot1, Fot2, Fot3, FotX and FotY display a substrate preference for oligopeptides mainly containing hydrophobic amino acids (Ala, Val, Leu, Ile, Met, Phe), Tyr (polar, uncharged) or His (positively charged), with distinct levels of consumption. Although Marsit et al. [7,8] showed that the oligopeptide fraction from grape must consumed by Fot1–Fot2-expressing strains was particularly rich in Glu/Gln, we characterized here oligopeptide preferences from phenotype microarrays. The range of di- and tripeptides offered by phenotype microarrays is not necessarily representative of the oligopeptides present in grape must, which additionally includes even longer oligopeptides.

In addition to hydrophobic amino acids, the preference of strains expressing Fot family members for oligopeptides containing Arg at the C-terminal position was remarkable. In their study of di- and tripeptide utilization by *S. cerevisiae*, Homann et al. [19] detected an unusual consumption of peptides with Arg in C-terminal position not due to Ptr2 or Dal5 in the vineyard-isolated strain RM8. The presence of Fot within the RM8 strain might therefore explain the preference of this strain for peptides with Arg in the C-terminal when Ptr2 and Dal5 were deleted. Interestingly, the *S. cerevisiae* di- and tripeptide transporter Ptr2 has similar preferences of oligopeptide composition, with a higher affinity for aromatic, branched or basic amino acids in the N-terminal position, also known as N-end rule dipeptides, and a lower affinity for negatively charged amino acids, i.e., Gly or Pro [28]. On the other hand, the allantoate permease Dal5, which is the main dipeptide transporter in some *S. cerevisiae* strains, preferably transports non-N-rule dipeptides [29].

Therefore, Dal5 and Ptr2 have complementary activities that allow *S. cerevisiae* to widen the spectrum of assimilable oligopeptides [19]. Considering that *S. cerevisiae* wine strain EC1118 has a non-functional Ptr2, it seems consistent that *FOT* acquisition from *T. microellipsoides* served to compensate for the lack of N-end rule oligopeptide consumption. However, Fot1–Fot2 in strain 59A, as well as EnvFot-F and EnvFot-A from unidentified eukaryotes have shown wider oligopeptide consumption than strains expressing Ptr2 or Dal5 [5], evidencing that *FOT* acquisition by *S. cerevisiae* wine strains not only fulfilled the need to compensate Ptr2 activity but also enlarged their ability to consume oligopeptides from the environment. Nonetheless, comparative studies on the presence of Fot, Pot/Ptr, Opt and Dal5 transporters among fungi are still required to better understand the evolution and biological relevance of oligopeptide transport in this kingdom.

Despite the general preference for N-end rule di- and tripeptides among strains with the distinct Fot members, levels of consumption did differ. The most extreme difference was found between FotX and FotY (Figure 1, rows 4 and 9 in the heatmap, respectively), with 74 di- and tripeptides consumed by the strain harboring FotX but not by FotY-expressing strain (Table S3). Considering the 91.87% of protein sequence identity between FotY and FotX (identity even higher between the other Fot members), the hypothesis is that Fot substrate specificity may depend on localized sequence differences. Fot1, which originated from gene conversions between *FOTX* and *FOT2Tm* from *T. microellipsoides* [7], displays a higher sequence identity with FotX (98.11% at protein level) than with Fot2 (94.66%); therefore, it was expected that Fot1 substrate preferences would be close to those of FotX. However, Fot1 oligopeptide preferences were closer to those of Fot2 (Figure 1, rows 5 and 6–7 in the heatmap, respectively), which indicates that the gene segment provided by *FOT2Tm* to *FOT1* might be crucial for substrate specificity, binding and transport. Additionally, we observed that 59 oligopeptides were only consumed by strains containing both Fot1 and Fot2 and not by those strains that had either Fot1 or Fot2 alone (Figure S2). These 59 oligopeptides were particularly rich in Gly and Trp at N- and C-termini, and Glu at the C-terminus. This striking difference between single Fot1 or Fot2 and Fot1–Fot2 together may indicate an interaction between these two transporters, such as oligomerization, with a potential effect on their transport properties; indeed, this phenomenon has been reported in other proton-coupled nutrient transporters located in the plasma membrane of plant cells [30]. In the filamentous fungus *Aspergillus nidulans*, dimerization of the H+-coupled uric acid–xanthine symporter UapA is suggested to have a role in transport activity [31]. Exploring the potential dimerization between Fot1 and Fot2 by bimolecular fluorescence complementation assays or with a yeast two-hybrid system could contribute to understanding Fot transport mechanisms and substrate specificity.

Levels of *FOT* gene expression were evaluated in two different *S. cerevisiae* wine strains at two key points of fermentation in two enological conditions. Normalized values of gene expression showed that, at the stationary phase, *FOT1* was more expressed than *FOT2* in strain 59A or *FOT3* in strain MTF2533, particularly in fermentation with synthetic must (Figure 3). It was unexpected to find a higher *FOT* expression in synthetic rather than in natural must, mainly because natural must contains Fot substrates as nitrogen sources, while synthetic must does not. Moreover, *FOT1* was more highly expressed after the depletion of assimilable nitrogen sources, which coincides with the entry in stationary phase. Previous studies have suggested that *FOT* genes were expressed from the beginning of fermentation since oligopeptide consumption by Fot took place before the consumption of some amino acids [8]. However, transcriptomic analyses on the commercial wine strain EC1118 during fermentation have shown that *FOT1* and *FOT2* are overexpressed in nitrogen-limiting conditions and repressed in nitrogen-rich media [32]. In addition, it was recently shown that *FOT1* displays higher transcriptional and translational levels in enological fermentations carried out in nitrogen-limiting conditions [33]. These results support the hypothesis that *FOT* genes are under NCR control, which prevents the expression of genes involved in the uptake of non-preferred nitrogen sources. Our findings on the presence of several Gln3- or Cup9-binding motifs in the promoter regions of *FOT1*, *FOT2/FOT2Tm*, *FOT3*, *FOTX* and *FOTY* (Table S5) further support this hypothesis. In this way, *FOT* expression would follow a pattern similar to that of the other genes coding for oligopeptide transporters in *S. cerevisiae*, viz. *PTR2*, *DAL5*, *OPT1* and *OPT2*, which are under NCR control and whose expression is induced upon the starvation of preferred nitrogen sources [1,32,33,34,35,36,37,38]. Transcriptomic analyses of the strains within the evaluated conditions of the present study by RNA-Seq would enable the comparative analysis of *FOT* expression and expression profiles of *GLN3*, other NCR regulators and genes reported to be under control of the NCR system.

Although current induction experiments conducted by Damon et al. (2011) [5] supported the hypothesis that Fot were plasma membrane transporters, proof was still lacking for their exact localization. In this work, we provide experimental evidence that Fot1 is localized in the plasma membrane in *S. cerevisiae* (Figure 4). During these experiments, we also observed a high instability of the C-terminal Fot1–GFP fusion. A possible explanation for this result lies in Fot secondary structure and membrane topology. Fot protein sequence analysis predicts a consensus membrane topology with an N-terminus located in the cytosol whereas the C-terminus faces yeast periplasm [5]. Under this assumption, GFP would be located at the surface of yeast cells expressing pFot1–GFP. Although there are examples of yeasts expressing GFP-fusion proteins at the extracellular side of the plasma membrane [39], there is also the assumption that GFP cannot emit fluorescence outside the cell due to structural instability, which may be due to proteolytic cleavage when the protein is exported [40]. However, since there are no experimental data available for the study of Fot protein structure, we cannot rule out other explanations for the weak signal observed for Fot1–GFP. The Fot1 C-terminal domain may be involved in regulatory processes that could be compromised, due to GFP interference in cells expressing the Fot1–GFP fusion. Indeed, in the sodium-coupled neutral amino acid transporter 2 Snat2, a membrane transporter with the same membrane topology as predicted for Fot [41], the C-terminal domain is a key element in the membrane voltage regulation for a normal amino acid translocation activity [42]. In the same way, the C-termini of Fot family members could be involved in the targeting at the plasma membrane or in membrane stabilization.

In conclusion, with this work we have increased our knowledge on the Fungal Oligopeptide Transporters family at three different levels: substrate specificity, subcellular localization in yeast and gene expression. Linking the differences found in oligopeptide consumption between Fot family members with their sequence divergence can reveal crucial protein motifs for substrate recognition, binding and transport. Further studies on the regulatory pathways involved in Fot expression in *S. cerevisiae* wine strains can lead to a better understanding of nitrogen metabolism during enological fermentation and to an overall vision of the role of oligopeptides as nitrogen sources in natural environments.

## Figures and Tables

**Table 1:** Strains used in this study.

Strain	Genotype	Source/Reference
59A	*MATa ho amn1Δ::LOXP*	[7]
fot1fot2Δ	*MATa ho amn1Δ::LOXP fot1fot2Δ::KANMX4*	This study
opt1Δopt2Δdal5Δ	*MATa ho amn1Δ::LOXP opt1Δ opt2Δ dal5Δ*	This study
PepKO	*MATa ho amn1Δ::LOXP fot1fot2Δ::KANMX4 opt1Δ opt2Δ dal5Δ*	This study
PepKO-Fot1	*MATa ho amn1Δ::LOXP opt1Δ opt2Δ dal5Δ fot1fot2Δ::FOT1*	This study
PepKO-Fot2	*MATa ho amn1Δ::LOXP opt1Δ opt2Δ dal5Δ fot1fot2Δ::FOT2*	This study
PepKO-Fot3	*MATa ho amn1Δ::LOXP opt1Δ opt2Δ dal5Δ fot1fot2Δ::FOT3*	This study
PepKO-FotX	*MATa ho amn1Δ::LOXP opt1Δ opt2Δ dal5Δ fot1fot2Δ::FOTX*	This study
PepKO-FotY	*MATa ho amn1Δ::LOXP opt1Δ opt2Δ dal5Δ fot1fot2Δ::FOTY*	This study
PepKO-Fot2Tm	*MATa ho amn1Δ::LOXP opt1Δ opt2Δ dal5Δ fot1fot2Δ::FOT2Tm*	This study
PepKO-Fot1Fot2	*MATa ho amn1Δ::LOXP opt1Δ opt2Δ dal5Δ fot1fot2Δ::FOT1–FOT2*	This study
59A-GFP	*MATa ho amn1Δ* *::TEFp-GFP-ADH1-NATMX4*	[7]
MTF2533	*MATa ho::LOXP*	[12]

## Data Availability

Not applicable.

## Supplementary Materials

The following are available online at https://www.mdpi.com/article/10.3390/jof7110963/s1, Table S1: Primers used in this study. Table S2: Plasmids used in this study. Table S3: Level of consumption of each oligopeptide by the different strains tested. Table S4: Amino acid composition of N- and C-termini of oligopeptides consumed by Fot. Table S5: Binding motifs for transcription factors (TFs) found in *FOT* promoter regions. Figure S1: Sequence alignment of Fot protein sequences from *S. cerevisiae* wine and *T. microellipsoides*. Figure S2: Oligopeptides consumed by strains with Fot1 and Fot2 together but not singly. Figure S3: Fermentation kinetics and growth of strains 59A and MTF2533 for gene expression analyses. Figure S4: Superposition of GFP and FM4–64 fluorescence channels in strains 59A-GFP and 59A containing pGFP–Fot1 or pFot1–GFP. References [43,44] are cited in Supplementary Table S2.

## References

[1] Perry J.R., Basrai A.M., Steiner H.Y., Naider F., Becker J.M. (1994). Isolation and Characterization of a *Saccharomyces cerevisiae* Peptide Transport Gene. Mol. Cell. Biol..

[2] Nelissen B., De Wachter R., Goffeau A. (1997). Classification of all Putative Permeases and Other Membrane Plurispanners of the Major Facilitator Superfamily Encoded by the Complete Genome of *Saccharomyces cerevisiae*. FEMS Microbiol. Rev..

[3] Hauser M., Donhardt A.M., Barnes D., Naider F., Becker J.M. (2000). Enkephalins are Transported by a Novel Eukaryotic Peptide Uptake System. J. Biol. Chem..

[4] Bourbouloux A., Shahi P., Chakladar A., Delrot S., Bachhawat A.K. (2000). Hgt1p, a High Affinity Glutathione Transporter from the Yeast *Saccharomyces cerevisiae*. J. Biol. Chem..

[5] Damon C., Vallon L., Zimmermann S., Haider M.Z., Galeote V., Dequin S., Luis P., Fraissinet-Tachet L., Marmeisse R. (2011). A Novel Fungal Family of Oligopeptide Transporters Identified by Functional Metatranscriptomics of Soil Eukaryotes. ISME J..

[6] Novo M., Bigey F., Beyne E., Galeote V., Gavory F., Mallet S., Cambon B., Legras J.-L., Wincker P., Casaregola S. (2009). Eukaryote-to-Eukaryote Gene Transfer Events Revealed by the Genome Sequence of the Wine Yeast *Saccharomyces cerevisiae* EC1118. Proc. Natl. Acad. Sci. USA.

[7] Marsit S., Mena A., Bigey F., Sauvage F.-X., Couloux A., Guy J., Legras J.-L., Barrio E., Dequin S., Galeote V. (2015). Evolutionary Advantage Conferred by an Eukaryote-to-Eukaryote Gene Transfer Event in Wine Yeasts. Mol. Biol. Evol..

[8] Marsit S., Sanchez I., Galeote V., Dequin S. (2016). Horizontally Acquired Oligopeptide Transporters Favour Adaptation of *Saccharomyces cerevisiae* Wine Yeast to Oenological Environment. Environ. Microbiol..

[9] Duc C., Maçna F., Sanchez I., Galeote V., Delpech S., Silvano A., Mouret J.-R. (2020). Large-Scale Screening of Thiol and Fermentative Aroma Production during Wine Alcoholic Fermentation: Exploring the Effects of Assimilable Nitrogen and Peptides. Fermentation.

[10] Becerra-Rodríguez C., Marsit S., Galeote V. (2020). Diversity of Oligopeptide Transport in Yeast and Its Impact on Adaptation to Winemaking Conditions. Front. Genet..

[11] Ambroset C., Petit M., Brion C., Sanchez I., Delobel P., Guérin C., Chiapello H., Nicolas P., Bigey F., Dequin S. (2011). Deciphering the Molecular Basis of Wine Yeast Fermentation Traits Using a Combined Genetic and Genomic Approach. G3 Genes Genomes Gen..

[12] Coi A.L., Legras J.-L., Zara G., Dequin S., Budroni M. (2016). A Set of Haploid Strains Available for Genetic Studies of *Saccharomyces cerevisiaeflor* Yeasts. FEMS Yeast Res..

[13] Wang Y., Shirogane T., Liu D., Harper J., Elledge S.J. (2003). Exit from Exit: Resetting the Cell Cycle through Amn1 Inhibition of G Protein Signaling. Cell.

[14] Yvert G., Brem R.B., Whittle J., Akey J.M., Foss E., Smith E.N., Mackelprang R., Kruglyak L. (2003). Trans-Acting Regulatory Variation in *Saccharomyces cerevisiae* and the Role of Transcription Factors. Nat. Genet..

[15] Bely M., Sablayrolles J.-M., Barre P. (1990). Automatic Detection of Assimilable Nitrogen Deficiencies during Alcoholic Fermentation in Oenological Conditions. J. Ferment. Bioeng..

[16] Stovicek V., Borodina I., Forster J. (2015). CRISPR–Cas System Enables Fast and Simple Genome Editing of Industrial *Saccharomyces cerevisiae* Strains. Metab. Eng. Commun..

[17] Mans R., Van Rossum H.M., Wijsman M., Backx A., Kuijpers N.G., Broek M.V.D., Daran-Lapujade P., Pronk J., van Maris A., Daran J.-M. (2015). CRISPR/Cas9: A Molecular Swiss Army Knife for Simultaneous Introduction of Multiple Genetic Modifications in *Saccharomyces cerevisiae*. FEMS Yeast Res..

[18] Gietz R.D., Schiestl R.H. (2007). High-Efficiency Yeast Transformation using the LiAc/SS carrier DNA/PEG method. Nat. Protoc..

[19] Homann O.R., Cai H., Becker J.M., Lindquist S.L. (2005). Harnessing Natural Diversity to Probe Metabolic Pathways. PLoS Genet..

[20] Vida A.T., Emr S.D. (1995). A New Vital Stain for Visualizing Vacuolar Membrane Dynamics and Endocytosis in Yeast. J. Cell Biol..

[21] Schindelin J., Arganda-Carreras I., Frise E., Kaynig V., Longair M., Pietzsch T., Preibisch S., Rueden C., Saalfeld S., Schmid B. (2012). Fiji: An Open-Source Platform for Biological-Image Analysis. Nat. Methods.

[22] De Boer C.G., Hughes T.R. (2012). YeTFaSCo: A Database of Evaluated Yeast Transcription Factor Sequence Specificities. Nucleic Acids Res..

[23] Wickham H., Averick M., Bryan J., Chang W., McGowan L., François R., Grolemund G., Hayes A., Henry L., Hester J. (2019). Welcome to the Tidyverse. J. Open Source Softw..

[24] Galili T., O’Callaghan A., Sidi J., Sievert C. (2018). Heatmaply: An R Package for Creating Interactive Cluster Heatmaps for Online Publishing. Bioinformatics.

[25] Rubio-Texeira M., Van Zeebroeck G., Thevelein J. (2012). Peptides Induce Persistent Signaling from Endosomes by a Nutrient Transceptor. Nat. Chem. Biol..

[26] Hauser M., Narita V., Donhardt A.M., Naider F., Becker J.M. (2001). Multiplicity and Regulation of Genes Encoding Peptide Transporters in Saccharomyces cerevisiae. Mol. Membr. Biol..

[27] Wiles A.M., Cai H., Naider F., Becker J.M. (2006). Nutrient Regulation of Oligopeptide Transport in *Saccharomyces cerevisiae*. Microbiology.

[28] Ito K., Hikida A., Kawai S., Lan V.T.T., Motoyama T., Kitagawa S., Yoshikawa Y., Kato R., Kawarasaki Y. (2013). Analysing the Substrate Multispecificity of a Proton-Coupled Oligopeptide Transporter using a Dipeptide Library. Nat. Commun..

[29] Cai H., Hauser M., Naider F., Becker J.M. (2007). Differential Regulation and Substrate Preferences in Two Peptide Transporters of Saccharomyces cerevisiae. Eukaryot. Cell.

[30] Reinders A., Schulze W., Kühn C., Barker L., Schulz A., Ward J., Frommer W.B. (2002). Protein–Protein Interactions between Sucrose Transporters of Different Affinities Colocalized in the Same Enucleate Sieve Element. Plant Cell.

[31] Alguel Y., Cameron A.D., Diallinas G., Byrne B. (2016). Transporter Oligomerization: Form and Function. Biochem. Soc. Trans..

[32] Duc C., Pradal M., Sanchez I., Noble J., Tesnière C., Blondin B. (2017). A Set of Nutrient Limitations Trigger Yeast Cell Death in a Nitrogen-Dependent Manner during Wine Alcoholic Fermentation. PLoS ONE.

[33] Devia J., Bastías C., Kessi E., Villarroel C.A., De Chiara M., Cubillos F., Liti G., Martínez C., Salinas F. (2020). Transcriptional Activity and Protein Levels of Horizontally Acquired Genes in Yeast Reveal Hallmarks of Adaptation to Fermentative Environments. Front. Genet..

[34] Bon E., Carvajal E., Stanbrough M., Rowen D., Magasanik B. (1997). Asparaginase II of *Saccharomyces cerevisiae*. Appl. Biochem. Biotechnol..

[35] Lubkowitz M.A., Barnes D., Breslav M., Burchfield A., Naider F., Becker J.M. (1998). *Schizosaccharomyces pombe* isp4 encodes a Transporter Representing a Novel Family of Oligopeptide Transporters. Mol. Microbiol..

[36] Magasanik B., Kaiser A.C. (2002). Nitrogen Regulation in *Saccharomyces cerevisiae*. Gene.

[37] Island M.D., Naider F., Becker J.M. (1987). Regulation of Dipeptide Transport in *Saccharomyces cerevisiae* by Micromolar Amino Acid Concentrations. J. Bacteriol..

[38] Rai R., Genbauffe F., Lea H.Z., Cooper T.G. (1987). Transcriptional Regulation of the DAL5 Gene in *Saccharomyces cerevisiae*. J. Bacteriol..

[39] Bleve G., Zacheo G., Cappello M.S., Dellaglio F., Grieco F. (2005). Subcellular Localization and Functional Expression of the Glycerol Uptake Protein 1 (GUP1) of *Saccharomyces cerevisiae* tagged with Green Fluorescent Protein. Biochem. J..

[40] Feilmeier B., Iseminger G., Schroeder D., Webber H., Phillips G.J. (2000). Green Fluorescent Protein Functions as a Reporter for Protein Localization in Escherichia coli. J. Bacteriol..

[41] Hyde R., Cwiklinski E.L., MacAulay K., Taylor P.M., Hundal H.S. (2007). Distinct Sensor Pathways in the Hierarchical Control of SNAT2, a Putative Amino Acid Transceptor, by Amino Acid Availability. J. Biol. Chem..

[42] Zhang Z., Albers T., Fiumera H.L., Gameiro A., Grewer C. (2009). A Conserved Na+ Binding Site of the Sodium-coupled Neutral Amino Acid Transporter 2 (SNAT2). J. Biol. Chem..

[43] Gueldener U. (2002). A Second Set of loxP Marker Cassettes for Cre-Mediated Multiple Gene Knockouts in Budding Yeast. Nucleic Acids Res..

[44] Breslow D., Cameron D.M., Collins S., Schuldiner M., Stewart-Ornstein J., Newman H.W., Braun S., Madhani H., Krogan N.J., Weissman J.S. (2008). A Comprehensive Strategy Enabling High-Resolution Functional Analysis of the Yeast Genome. Nat. Methods.

