# Supplementary material for: Yeast Plasma Membrane Fungal Oligopeptide Transporters Display Distinct Substrate Preferences despite Their High Sequence Identity
Source: J Fungi (Basel). 2021 Nov 12;7(11):963. doi: 10.3390/jof7110963 (PMC8625066; doi:10.3390/jof7110963)
Supplement: Supplementary file 1 [file jof-07-00963-s001.zip › Figures_S1-S2-S3-S4.pdf]

SUPPLEMENTARY INFORMATION

```
Fot3    MSKLVPIASPNLSMMTSRAYDEAKK---EKGSQGTIDHEKGEYVDISGREIISSESEKHGRQLPLQAYVHYAEIQREFER [80]
FotX    MSKLVPIASPNLSMMTSRAYDEAKK---EKGSQGTIDHEKGEYVDISGREIISSESEKHGRQLPLQAYVHYAEIQREFER [80]
Fot1    MSKLVPIASPNLSMMTSRAYDEAKK---EKGSQGTIDHEKGEYVDISGREIISSESEKHGRQLPLQAYVHYAEIQREFER [80]
Fot2Tm  MSKLIPIASPALSMMTSRAYDEVKNENVEKGEQGSVD-EKGEYVDVLGREIISSETEKHGN-LPLQAYVHYAGIQREFER [80]
Fot2    MSKLIPIASPALSMMTSRAYDEVKNENVEKGEQGSVD-EKGEYVDVLGREIISSETEKHGN-LPLQAYVHYAGIQREFER [80]
FotY    MSKLIPIASPTLSMMTSGAYDDVKK---ERAEQSSAS-EKGEYVDVSGREIISIESEKHGK-IPLQAYFHYADIQREFER [80]

Fot3    ERDEDGLYAVQREQMYTDPHSNVSPSKLDSQRMLRIAKAYSVFFLITTDILGPSNAPYAVAQMGWVPGVILYVIFGVAAA [160]
FotX    ERDEDGLYAVQREQMYTDPHSNVSPSKLDSQRMLRIAKAYSVFFLITTDILGPSNAPYAVAQMGWVPGVILYVLFGVAAA [160]
Fot1    ERDEDGLYAVQREQMYTDPHSNVSPSKLDSQRMLRIAKAYSVFFLITTDILGPSNAPYAVAQMGWVPGVILYVLFGVAAA [160]
Fot2Tm  ERDEDGLYAVQREQMYTDPHSNVSPSKLDSQRMLRIAKAYSVFFLITTDILGPSNAPYAVAQMGWVPGVILYVIFGVAAA [160]
Fot2    ERDEDGLYAVQREQMYTDPHSNVSPSKLDSQRMLRIAKAYSVFFLITTDILGPSNAPYAVAQMGWVPGVILYVIFGVAAA [160]
FotY    ESDEDGLYAAQREQMYTDPHSNVSPTKLDSQRMLRIAKAYSVFFLITTDILGPANAPYAVAQMGWVPGVILYVVFGVAAA [160]

Fot3    FGGWLLNFCFCKVDSNNYPIRTFSDLAARVVVPWFRYPFGLLQFIQMILNCGLLLLSTAQSVSQMLVVNRGGDHFCFTVD [240]
FotX    FGGWLLNFCFCKVDSNNYPIRTFSDLAARVVAPWFRYPFGLLQFIQMILNCGLILLSTAQSVSQMLVVNRGGDDFCFTVD [240]
Fot1    FGGWLLNFCFCKVDSNNYPIRTFSDLAARVVAPWFRYPFGLLQFIQMILNCGLILLSTAQSVSQMLVVNRGGDDFCFTVD [240]
Fot2Tm  FGGWLLNFCFCKVDSNNYPIRTFSDLAARVVAPWFRYPFGLLQFIQMILNCGLLLLSTAQSVSQMLVVNRGGDHFCFTVD [240]
Fot2    FGGWLLNFCFCKVDSNNYPIRTFSDLAARVVAPWFRYPFGLLQFIQMILNCGLLLLSTAQSVSQMLVVNRGGDHFCFTVD [240]
FotY    VGGWLLNFCFCKVDSNNYPIRTFSDLAARVVAPWFRYPFGLLQFIQMILNCGLLLLSTAQSVSQMLVVNRGGDHFCFTVD [240]

Fot3    ILVWGLLCMIMGQIRSLGRFAHIANSAVWMNIAICIITMVGVAVGGPYYGIFEQYGQGAPYFQPSSYIPLPIKHYAIVPG [320]
FotX    ILVWGLLCMILGQIRSLGRFAHIANSAVWMNIAICIITMVGVAVGGPYYGIFEQYGQGAPYFQPSSYIPLPIKQYAIVPG [320]
Fot1    ILVWGLLCMILGQIRSLGRFAHIANSAVWMNIAICIITMVGVAVGGPYYGIFEQYGQGAPYFQPSSYIPLPIKQYAIVPG [320]
Fot2Tm  ILVWGLLCMIMGQIRSLGRFAHIANSAVWMNIAICIITMVGVALGGPYYGIFEQYGQGAPYFQPSSYIPLPIKQYAIVPG [320]
Fot2    ILVWGLLCMIMGQIRSLGRFAHIANSAVWMNIAICIITMVGVALGGPYYGIFEQYGQGAPYFQPSSYIPLPIKQYAIVPG [320]
FotY    ILVWGLLCMIMGQIRSLGRFAHIANSAVWMNIAVCIITMVGVAVGGPYYGIFEQYGQGAPYFQPSSYIPLPIKQYAIVPG [320]

Fot3    NISDKIAGMNNMVFAWGGATIFCEVMAEMKRPMDFWKGMLCAQSLILVVYLFYGLFVYAYNGQFSYVTANMAIGSIGLQN [400]
FotX    NISDKIAGMNNMVFAWGGATIFCEVMAEMRRPMDFWKGMLCAQSLILVVYLFYGLFVYAYNGQFSYVTANMAIGSIGLQN [400]
Fot1    NISDKIAGMNNMVFAWGGATIFCEVMAEMKRPMDFWKGMLCAQSLILVVYLFYGLFVYAYNGQFSYVTANMAIGSIGLQN [400]
Fot2Tm  NISDKIAGMNNMVFAWGGATIFCEVMAEMRRPMDFWKGMLCAQSLILVVYLFYGLFVYAYNGQFSYVTANMAIGSIGLQN [400]
Fot2    NISDKIAGMNNMVFAWGGATIFCEVMAEMRRPMDFWKGMLCAQSLILVVYLFYGLFVYAYNGQFSYVTANMAIGSIGLQN [400]
FotY    NISDKISGMNNMVFAWGGATIFCEVMAEMKRPMDFWKGMLCAQSLILVVYLFYGLFVYAYNGQFSYVTANMGIGSIALQN [400]

Fot3    AGNVLSIISGIIAMVLYGNIGIKVVYQGFLVTDFNFPSLTSRKGTFAWGGFVILYWAVAYILGTAIPSISALVGIVGAFC [480]
FotX    AGNVLSIISGIIAMVLYGNIGIKVVYQGFLVTDFNFPSLTSRKGTFAWGGFVILYWAVAYILGTAIPSISALVGIVGAFC [480]
Fot1    AGNVLSIISGIIAMVLYGNIGIKVIYQGFLVTDFNFPSLTSRKGTFAWAGFVVVYWAIAYILGTAIPSISALVAIVGAFC [480]
Fot2Tm  AGNVLTIITGIIAMVLYGNIGIKVIYQGFLVTDFNFPSLTSRKGTFAWAGFVVVYWAIAYILGTAIPSISALVAIVGAFC [480]
Fot2    AGNVLTIITGIIAMVLYGNIGIKVIYQGFLVTDFNFPSLTSRKGTFAWAGFVVVYWAIAYILGTAIPSISALVAIVGAFC [480]
FotY    AGNVLTIITGIIAMVLYGNIGIKVIYQGFLVTDFKFPSLTSRKGTIAWAGFVILYWAVAYILGTAIPSISALVGIVGAFC [480]

Fot3    ILNFSYTFPFLFGFCLLCRQDAALADNFDAKTLTVEKADSYREWSRWKRALGYGGIYRTSIKVSLFLLFLASLATCGLCS [560]
FotX    ILNFSYTFPFLFGFCLLCRQDAALADNFDAKTLTVEKADSYREWSRWKRALGYGGIYRTSIKVSLFLLFLASLATCGLCS [560]
Fot1    ILNFSYTFPFLFGFCLLCRQDAALADNFDAKTLTVEKADSYRSWSRWKRALGYGGTYRILIKVSLFLLFLASLATCGLCS [560]
Fot2Tm  ILNFSYTFPFLFGFCLLCRQDAALADNFDAKTLTVEKADSYRSWSRWKRALGYGGTYRILIKVSLFLLFLASLATCGLCS [560]
Fot2    ILNFSYTFPFLFGFCLLCRQDAALADNFDAKTLTVEKADSYRSWSRWKRALGYGGTYRILIKVSLFLLFLASLATCGLCS [560]
FotY    ILNFSYTFPFLFGFCLLWRQDAALADHFDPKTLSIERTDSYRNWSRWKRALGYGGIRRTAIKVSLFLLFLASLATCGLCS [560]

Fot3    YSAISGAIAVYQTNPAQPFTCTSPVA [586]
FotX    YSAISGAIAVYQTNPAQPFTCTSPVA [586]
Fot1    YSAISGAIAVYQTNPAQPFTCTSPVA [586]
Fot2Tm  YSAISGAIAVYQTNPAQPFTCTSPVA [586]
Fot2    YSAISGAIAVYQTNPAQPFTCTSPVA [586]
FotY    YSAISGAIEVYQTNPAQPFTCTSPVA [586]
```

Figure S1. Sequence alignment of Fot protein sequences from *S. cerevisiae* wine and *T. microellipsoides*. Sequences were aligned with MUSCLE using a BLOSUM62 matrix and a gap extension and gap open penalties of respectively -1 and -12. Variable sites are indicated in orange. Sequences are sorted according to their pairwise identity.

A)

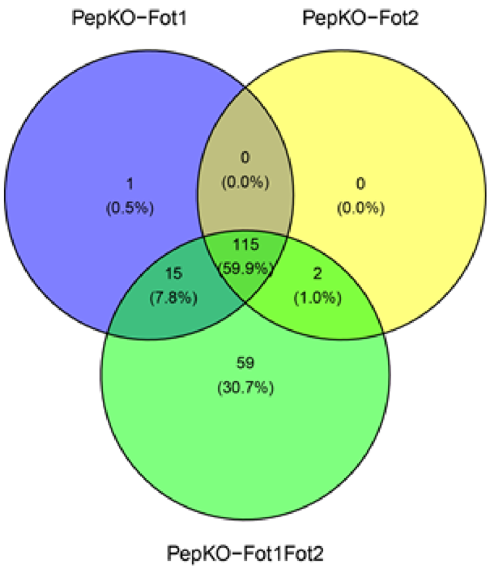

B)

4 (21/59)	3 (9/59)	2 (21/59)	1 (8/59)
Ala-Pro	Leu-Asp	Ala-Glu	Ala-Asp
Ala-Ser	Leu-Glu	Ala-Gly	Asp-Trp
Arg-Trp	Met-Pro	Arg-Lys	Gly-Leu
Glu-Ala	Phe-Pro	Arg-Tyr	Gly-Thr
Glu-Val	Ser-Pro	Asn-Glu	Lys-Phe
Gly-Gly-Phe	Ser-Ser	Asp-Phe	Lys-Trp
Gly-Phe	Thr-Glu	Gln-Glu	Trp-Asp
Gly-Tyr	Thr-Gly	Gln-Gly	Trp-Trp
Gly-Val	Tyr-Gly	Gly-Ala	
His-Asp		Gly-Gln	
His-Glu		Gly-Gly-Ile	
His-Gly		Gly-Gly-Leu	
Ile-Gly		Gly-Met	
Leu-Gly		Leu-b-Ala	
Leu-Gly-Gly		Phe-b-Ala	
Met-Glu		Pro-Trp	
Met-Gly		Ser-Gly	
Trp-Val		Thr-Pro	
Val-Asp		Trp-Leu	
Val-Gly		Trp-Ser	
Val-Pro		Val-Glu	

C)

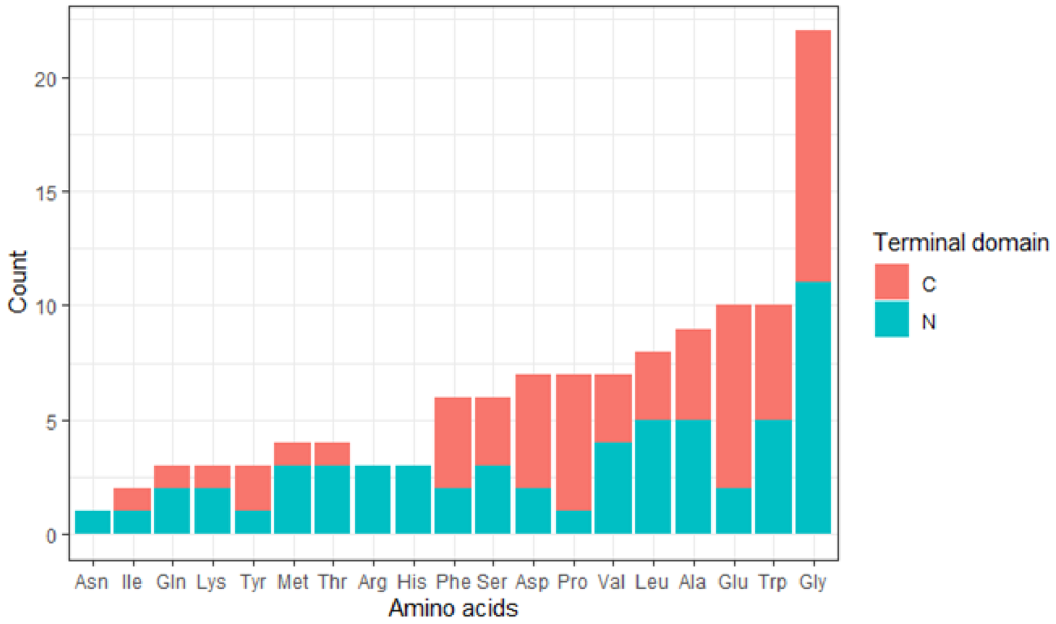

Figure S2. Oligopeptides consumed by strains with Fot1 and Fot2 together but not singly. A) Venn diagram with frequencies of oligopeptides consumed by Fot1, Fot2 and Fot1Fot2 containing strains. **B)** Table headings correspond to the level of oligopeptide consumption category. Level 4: 80-100% of positive control consumption; 3: 60-80%; 2: 40-60%; 1: 20-40%. **C)** Amino acid frequency in N- and C-terminus of oligopeptides.

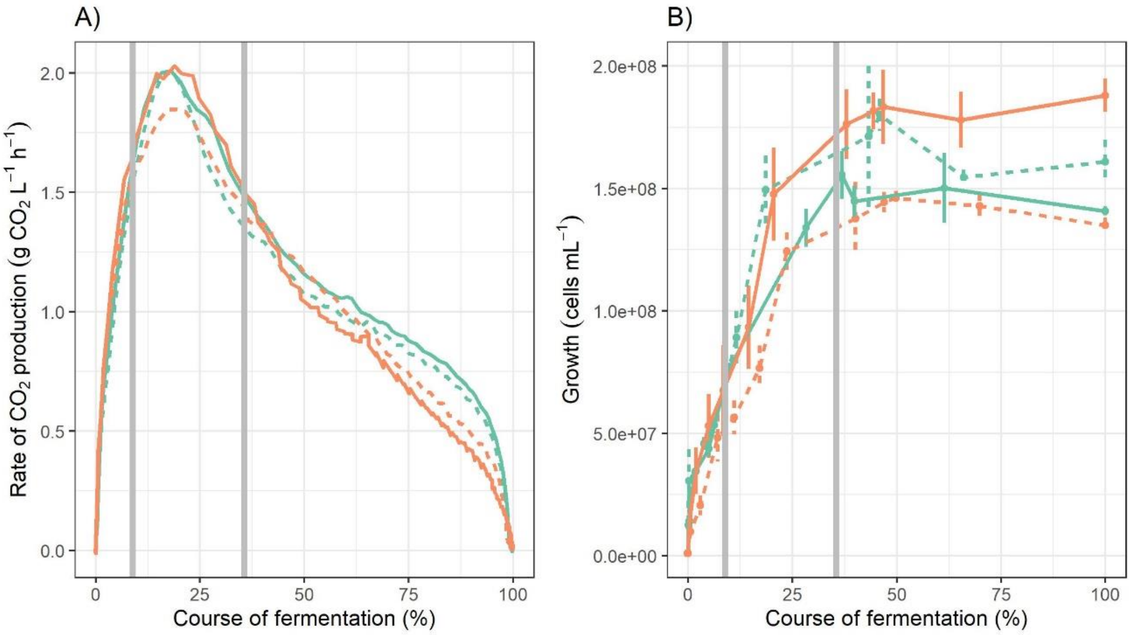

Figure S3. Fermentation kinetics and growth of strains 59A and MTF2533 for gene expression analyses. Grey vertical lines indicate 10% and 40% of fermentation. Fermentations on natural grape must are represented with a continuous line, and discontinuous lines represent fermentations in synthetic must, with strain 59A in green and MTF2533 in orange. **A)** Fermentation kinetics and **B)** cell growth throughout fermentation. n =3.

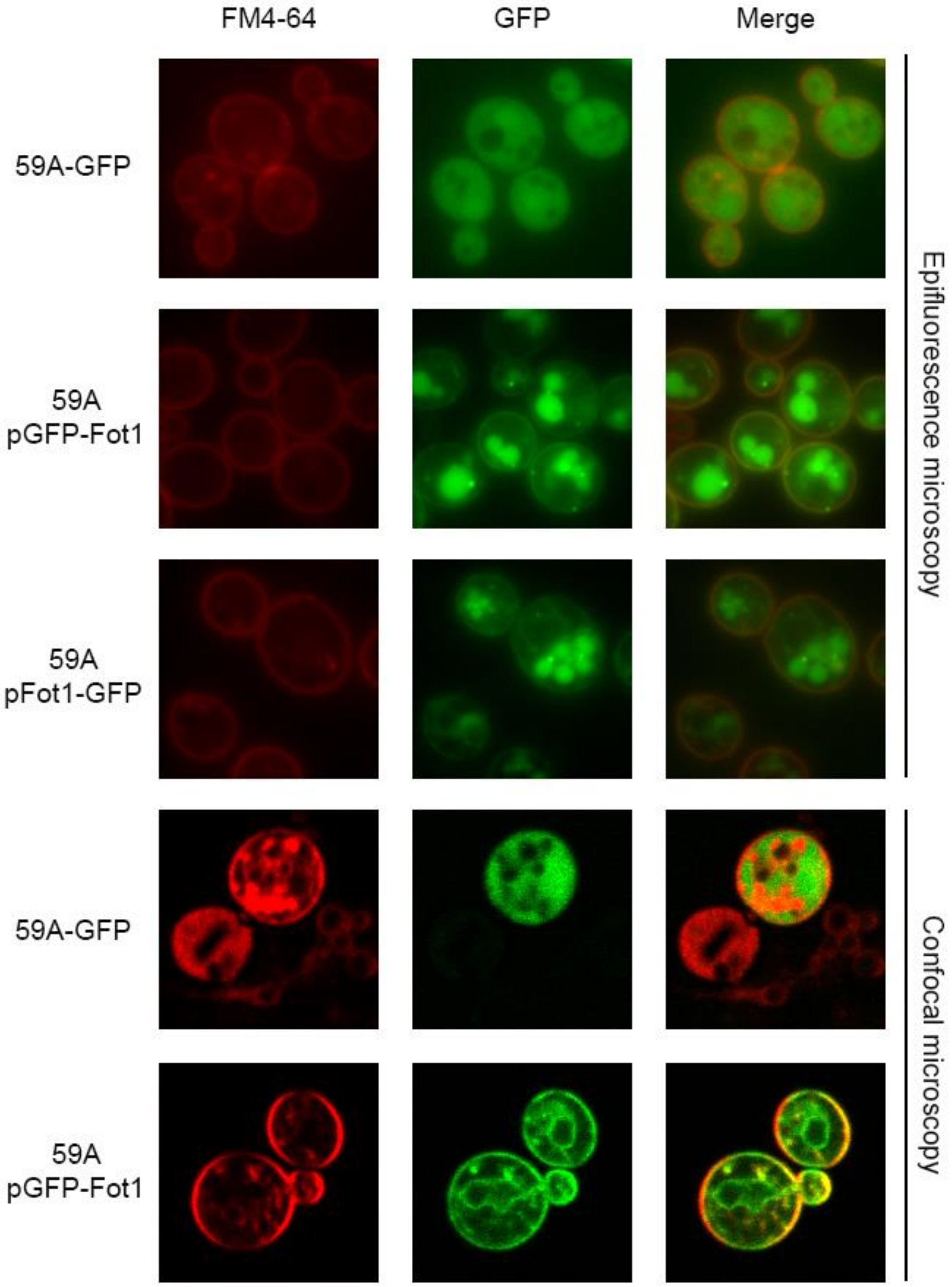

Figure S4. Superposition of GFP and FM4-64 fluorescence channels in strains 59A-GFP and 59A containing pGFP-Fot1 or pFot1-GFP.
