# Supplementary material for: Yeast Plasma Membrane Fungal Oligopeptide Transporters Display Distinct Substrate Preferences despite Their High Sequence Identity
Source: J Fungi (Basel). 2021 Nov 12;7(11):963. doi: 10.3390/jof7110963 (PMC8625066; doi:10.3390/jof7110963)
Supplement: Supplementary file 1 [file jof-07-00963-s001.zip › Table_S1-S2-S3-S4.pdf]

Table S1. Primers used in this study.

Primer	Sequence
Deletion of *FOT1-FOT2* in strain 59A	
DelFotYap_R	ACCATGGGATTTCCGTCGTCCTGTGATGGCAACTGCCTTATTTAGAGCTTGACGGGGAAA
DelFotYap_F	AAAAATTTATTACTGAAGATGTCAAAGCTCATCCCTATCGGATCGGAATTCATGAGTGTGTC
Amplification of KANMX4 with flanking regions of *FOT1-FOT2* in strain 59A	
DelFotKan_F	AAAAATTTATTACTGAAGATGTCAAAGCTCATCCCTATCGGCATAGGCCACTAGTGGATCTG
DelFotKan_R	ACCATGGGATTTCCGTCGTCCTGTGATGGCAACTGCCTTATTCGTACGCTGCAGGTCGAC
Verification of *FOT1-FOT2* substitution by the *KANMX4* cassette	
CDFOT_F	TATATCCATTCAAGTCCCTC
CDFOT_R	GTGTCAATATTCTGGTTCAC
KAN_F	TGGGTAAGGAAAAGACTCACG
KAN_R	CTCATCGAGCATCAAATGAAA
Construction of plasmids containing Cas9 guide sequences	
6005	GATCATTTATCTTTCACTGCGGAGAAGTTT
targetDAL5	TGCTCAATACTGGAAGACAGGTTTTAGAGCTAGAAATAGCAAGTTAAAATAAGG
opt1target_1	GAAAACTACCACAAACACCGGTTTTAGAGCTAGAAATAGCAAGTTAAAATAAGG
opt2target_1	TGATGGTTATAGTCCAACAGGTTTTAGAGCTAGAAATAGCAAGTTAAAATAAGG
Kan_target4	CGCTGGCAGTGTTCCTGCGCGTTTTAGAGCTAGAAATAGCAAGTTAAAATAAGG
Amplification of repair fragment for *DAL5* deletion	
DAL5rf.FW	GAGCCAGTTCTAAGCACGGT
DAL5rf.RV	CCCGAGGAATTCGCACTGAT
Amplification of repair fragment for *OPT1* deletion	
OPT1repfrag.FW	TACAGGAGTATGAATGTGCTTAGCA
OPT1repfrag.RV	CGACTGTGTGGCAGAAAATAAC
Amplification of repair fragment for *OPT2* deletion	
OPT2rf.FW	CACATCTAGCTTATTGCCACTGTCT
OPT2rf.RV	GCCTGAATTGACACTTGAGGATTC

Amplification of *FOT* genes with flanking regions for their integration in substitution of *KANMX4* cassette

Fot1L.fw	ATTAGTTATATTTTATAGATCTGCTTGCCATCTTGCACAATATATCCATTCAAGTCCCTCACCATGGGATTTCCGTCGTCCTGTGATGGCAACTGCCTTA
Fot1-3L.rv	GTAAGGACAGATACCTCAATTTCAAATAGAAACTATAGTTCTTGCTTAACGATAACAGTCTATTCCATTTGACTGAAGAAAAAAAAATTTATTACTGA AGATGTCAAAACTTGTCCCTAT
Fot2L.fw	ATTAGTTATATTTTATAGATCTGCTTGCCATCTTGCACAATATATCCATTCAAGTCCCTCACCATGGGATTTCCGTCGTCCTGTGATGGCAACTGCCTTA TCAGGCCACAGGAGAAGTGC
Fot2L.rv	GTAAGGACAGATACCTCAATTTCAAATAGAAACTATAGTTCTTGCTTAACGATAACAGTCTATTCCATTTGACTGAAGAAAAAAAAATTTATTACTGA AGATGTCAAAGCTCATCCCTAT
FotYL.fw	ATTAGTTATATTTTATAGATCTGCTTGCCATCTTGCACAATATATCCATTCAAGTCCCTCACCATGGGATTTCCGTCGTCCTGTGATGGCAACTGCCTTA TCAGGCGACAGGAGAAGTAC
FotYL.rv	GTAAGGACAGATACCTCAATTTCAAATAGAAACTATAGTTCTTGCTTAACGATAACAGTCTATTCCATTTGACTGAAGAAAAAAAAATTTATTACTGA AGATGTCAAAACTTATCCCTAT

Construction of plasmids by Gibson assembly

pRS316_back.FW	AATTCACCAATTCGTTCATTAGCAAAAGGCCAGCAAAAGG
pRS316_back.RV	CCGGGTACCGAGCTCGAATTTAGGACGGATCGCTTGCCTG
yep352-G418.FW	CAGGCAAGCGATCCGTCCTAAATTCGAGCTCGGTACCCGG
yep352-G418.RV	AAGTATACGGCCCCATATATGGCCAGTGCCAAGCTTGCAT
pFA6a-TEFp-GFP.FW	ATGCAAGCTTGGCACTGGCCATATATGGGGCCGTATACTTAC
pFA6a-TEFp-GFP.RV	ATAGGGACAAGTTTTGACATTTTGTATAGTTCATCCATGCCA
Gib-GFP-FOT1.FW	GCATGGATGAACTATACAAAATGTCAAAACTTGTCCCTATCG
pr-Nter-GFP-FOT1.RV	CCTTTTGCTGGCCTTTTGCTAATGAACGAATTGGTGAATTAGC
yep352_back.FW	AATTCACCAATTCGTTCATTATCTATTGCATAGGTAATCTTGCACGTC
yep352_back.RV	AAGTATACGGCCCCATATATAAAGCCGCCGTCCCGTCAAG
yep_TEFp-GFP.FW	CTTGACGGGACGGCGGCTTTATATATGGGGCCGTATACTTAC
yep_GFP-FOT1.RV	AGATTACCTATGCAATAGATAATGAACGAATTGGTGAATTAGC

Primers for qPCR

QFOT1-X-Fw	GCGGTTGGTTGTTGAACTTT
QFOT1-X-Rv	GGGCAGTGCTCAGAAGAATC
QFOT2-Fw	CGAGGGCTTATGACGAGGTA
QFOT2-Rv	GATCCCAGCGTAGTGGACAT
QFOT3-Fw	GCGAGTGGTCTAGGTGGAAG
QFOT3-Rv	CACAGGAGAAGTGCAGGTGA
QFOTY-Fw	AAGCGTCCAATGGATTTTTG
QFOTY-Rv	ATTGCCAGCATTTTGTAGGG
QSCR1-Fw	CTTTCTGGTGGGATGGGATA
QSCR1-Rv	TTTACGACGGAGGAAAGACG

Table S2. Plasmids used in this study.

Name	Genotype/Features	Use	Reference
pUG6		Amplification of the *KANMX4* cassette	[43]
pCfB2513	*TEF1p-Cas9-CYC1t-2-loxP-hphMX*	Transformation of yeast with Cas9-containing plasmid; selection against hygromycin.	[16]
pMEL15-dal5	*2µm NATNT2 gRNA-DAL5*	Contains the guide sequence for deletion of *DAL5*; selection against nourseothricin.	[17]; This study
pMEL15-opt1	*2µm NATNT2 gRNA-OPT1*	Contains the guide sequence for deletion of *OPT1*; selection against nourseothricin.	[17]; This study
pMEL15-opt2	*2µm NATNT2 gRNA-OPT2*	Contains the guide sequence for deletion of *OPT2*; selection against nourseothricin.	[17]; This study
pMEL15-kan4	*2µm NATNT2 gRNA-KANMX4*	Contains the guide sequence for deletion of *KANMX4* cassette; selection against nourseothricin.	[17]; This study
pEX-A128-DAL5rf	pEX-A128	Contains the repair fragment sequence for *DAL5* deletion	Eurofins Genomics
pEX-A128-OPT1repfrag	pEX-A128	Contains the repair fragment sequence for *OPT1* deletion	Eurofins Genomics
pEX-A128-OPT2rf	pEX-A128	Contains the repair fragment sequence for *OPT2* deletion	Eurofins Genomics
pRS316	*CEN/ARS URA3 AgTEF1p-hphMX-AgTEF1t*		ATCC
YEp352-G418	*2µm URA3 PGKp-G418-PGKt*	Construction of pGFP-Fot1 and pFot1-GFP plasmids	ATCC
pFA6a	pFA6a-*TEF2Pr-eGFP-ADH1-NatMx4*		[44]
pGFP-Fot1	*CEN/ARS PGKp-G418- AgTEF1p-GFP-FOT1*	Contains *GFP-FOT1* fusion with *GFP* in *FOT1*'s N-terminus, CEN/ARS as origin of replication; selection against G418.	This study
pFot1-GFP	*CEN/ARS PGKp-G418- AgTEF1p-FOT1-GFP*	Contains *FOT1-GFP* fusion with *GFP* in *FOT1*'s C-terminus, CEN/ARS as origin of replication; selection against G418.	This study
yep-Nterm-*GFP-FOT1*	*2µm PGKp-G418- AgTEF1p-GFP-FOT1*	Contains *GFP-FOT1* fusion with *GFP* in *FOT1*'s N-terminus, 2µm as origin of replication; selection against G418.	This study
yep-Cterm-*FOT1-GFP*	*2µm PGKp-G418- AgTEF1p-FOT1-GFP*	Contains *FOT1-GFP* fusion with *GFP* in *FOT1*'s C-terminus, 2 µm as origin of replication; selection against G418.	This study

Table S3. Level of consumption of each oligopeptide by the different strains tested. Consumption was measured as values of Area Under the Curve (AUC). Levels of consumption were stablished based on the consumption value of L-Glutamine, positive control in each plate; 0, 0-20% of consumption on L-Gln; 1, 21-40%; 2, 41-60%; 3, 61-80%; 4, 81-100%; 5, >100%.

	59A (wt)	opt1Δ opt2Δ Δdal5	fot1fot2Δ	PepTrKO ::*FOT1*	PepTrKO ::*FOT1-FOT2*	PepTrKO ::*FOT2*	PepTrKO ::*FOT2Tm*	PepTrKO ::*FOT3*	PepTrKO ::*FOTX*	PepTrKO ::*FOTY*	PepTrKO
Ala-Ala	5	4	5	3	4	3	3	2	4	0	0
Ala-Ala-Ala	4	4	3	4	4	3	3	4	4	2	0
Ala-Arg	5	5	2	4	4	4	4	4	5	4	0
Ala-Asn	5	5	5	1	4	0	1	1	4	0	0
Ala-Asp	2	2	0	0	1	0	0	0	0	0	0
Ala-Gln	4	4	4	3	4	2	2	2	4	0	0
Ala-Glu	4	2	4	0	2	0	0	0	0	0	0
Ala-Gly	4	3	3	0	2	0	0	0	0	0	0
Ala-His	4	4	4	4	4	4	4	4	4	4	0
Ala-Ile	5	4	1	4	4	4	3	3	4	2	0
Ala-Leu	4	4	4	4	4	2	3	2	4	0	0
Ala-Lys	3	2	0	1	2	1	1	1	2	1	0
Ala-Met	4	4	5	4	4	4	3	1	4	4	0
Ala-Phe	5	5	0	4	4	4	4	0	4	4	0
Ala-Pro	5	4	0	0	4	0	0	0	0	0	0
Ala-Ser	5	4	5	0	4	0	0	0	2	0	0
Ala-Thr	4	4	4	3	4	4	4	4	4	0	0
Ala-Trp	5	4	0	3	4	1	2	0	4	0	0
Ala-Tyr	5	4	0	4	4	4	4	1	4	4	0
Ala-Val	5	4	0	4	4	4	3	4	4	2	0
Arg-Ala	0	0	0	0	0	0	0	0	0	0	0
Arg-Arg	5	4	2	3	4	2	1	1	4	0	0
Arg-Asp	0	0	0	0	0	0	0	0	0	0	0
Arg-Gln	0	0	0	0	0	0	0	0	0	0	0
Arg-Glu	0	0	0	0	0	0	0	0	0	0	0
Arg-Ile	0	0	0	0	0	0	0	0	0	0	0
Arg-Leu	2	0	2	0	0	0	0	0	0	0	0
Arg-Lys	4	3	0	0	2	0	0	0	0	0	0
Arg-Met	1	0	1	0	0	0	0	0	0	0	0
Arg-Phe	0	0	0	0	0	0	0	0	0	0	0
Arg-Ser	1	0	1	0	0	0	0	0	0	0	0
Arg-Trp	5	4	2	0	4	0	0	0	2	0	0
Arg-Tyr	4	2	0	0	2	0	0	0	0	0	0
Arg-Val	2	0	1	0	0	0	0	0	0	0	0
Asn-Glu	2	1	0	0	2	0	0	0	0	0	0

	59A (wt)	opt1Δ opt2Δ Δdal5	fot1fot2Δ	PepTrKO ::*FOT1*	PepTrKO ::*FOT1-FOT2*	PepTrKO ::*FOT2*	PepTrKO ::*FOT2Tm*	PepTrKO ::*FOT3*	PepTrKO ::*FOTX*	PepTrKO ::*FOTY*	PepTrKO
Asn-Val	5	4	0	4	4	4	4	4	4	4	0
Asp-Ala	0	0	0	0	0	0	0	0	0	0	0
Asp-Asp	0	0	0	0	0	0	0	0	0	0	0
Asp-Gln	0	0	0	0	0	0	0	0	0	0	0
Asp-Glu	0	0	0	0	0	0	0	0	0	0	0
Asp-Gly	0	0	0	0	0	0	0	0	0	0	0
Asp-Leu	0	0	0	0	0	0	0	0	0	0	0
Asp-Lys	1	0	0	0	0	0	0	0	0	0	0
Asp-Phe	4	2	0	0	2	0	0	0	0	0	0
Asp-Trp	2	1	0	0	1	0	0	0	0	0	0
Asp-Val	0	0	0	0	0	0	0	0	0	0	0
β-Ala-Ala	0	0	0	0	0	0	0	0	0	0	0
β-Ala-Gly	0	0	0	0	0	0	0	0	0	0	0
β-Ala-His	0	0	0	0	0	0	0	0	0	0	0
β-Ala-Phe	0	0	0	0	0	0	0	0	0	0	0
Cys-Gly	0	0	0	0	0	0	0	0	0	0	0
D-Ala-D-Ala	0	0	0	0	0	0	0	0	0	0	0
D-Ala-Gly	0	0	0	0	0	0	0	0	0	0	0
D-Ala-Gly-Gly	0	0	0	0	0	0	0	0	0	0	0
D-Ala-Leu	0	0	0	0	0	0	0	0	0	0	0
D-Leu-D-Leu	0	0	0	0	0	0	0	0	0	0	0
D-Leu-Gly	0	0	0	0	0	0	0	0	0	0	0
D-Leu-Tyr	0	0	0	0	0	0	0	0	0	0	0
γ-D-Glu-Gly	0	0	0	0	0	0	0	0	0	0	0
γ-Glu-Gly	1	1	2	2	1	1	1	2	3	0	1
Gln-Gln	5	5	1	2	4	0	1	0	4	0	0
Gln-Glu	2	2	0	0	2	0	0	0	0	0	0
Gln-Gly	4	2	0	0	2	0	0	0	0	0	0
Glu-Ala	4	3	0	0	4	0	0	0	0	0	0
Glu-Asp	0	0	0	0	0	0	0	0	0	0	0
Glu-Glu	0	0	0	0	0	0	0	0	0	0	0
Glu-Gly	0	0	0	0	0	0	0	0	0	0	0
Glu-Ser	0	0	0	0	0	0	0	0	0	0	0
Glu-Trp	5	3	0	2	2	0	0	0	3	0	0
Glu-Tyr	5	4	0	1	4	0	0	0	2	0	0
Glu-Val	5	4	0	0	4	0	0	0	1	0	0
Gly-Ala	5	1	5	0	2	0	0	0	0	0	0
Gly-Arg	5	5	0	4	4	4	4	4	4	4	0
Gly-Asn	5	0	5	0	0	0	0	0	0	0	0
Gly-Asp	0	0	0	0	0	0	0	0	0	0	0

	59A (wt)	opt1Δ opt2Δ Δdal5	fot1fot2Δ	PepTrKO ::*FOT1*	PepTrKO ::*FOT1-FOT2*	PepTrKO ::*FOT2*	PepTrKO ::*FOT2Tm*	PepTrKO ::*FOT3*	PepTrKO ::*FOTX*	PepTrKO ::*FOTY*	PepTrKO
Gly-Cys	4	0	3	0	0	0	0	0	0	0	0
Gly-D-Ala	0	0	0	0	0	0	0	0	0	0	0
Gly-D-Asp	0	0	0	0	0	0	0	0	0	0	0
Gly-D-Ser	0	0	0	0	0	0	0	0	0	0	0
Gly-D-Thr	0	0	0	0	0	0	0	0	0	0	0
Gly-D-Val	0	0	0	0	0	0	0	0	0	0	0
Gly-Gln	4	2	4	0	2	0	0	0	0	0	0
Gly-Glu	2	0	3	0	0	0	0	0	0	0	0
Gly-Gly	4	0	4	0	0	0	0	0	0	0	0
Gly-Gly-Ala	4	0	4	0	0	0	0	0	0	0	0
Gly-Gly-D-Leu	0	0	0	0	0	0	0	0	0	0	0
Gly-Gly-Gly	4	0	4	0	0	0	0	0	0	0	0
Gly-Gly-Ile	4	2	4	0	2	0	0	0	0	0	0
Gly-Gly-Leu	4	2	4	0	2	0	0	0	0	0	0
Gly-Gly-Phe	4	3	4	0	4	0	0	0	0	0	0
Gly-His	5	3	4	3	4	2	2	3	2	2	0
Gly-Ile	2	0	2	0	0	0	0	0	0	0	0
Gly-Leu	5	0	5	0	1	0	0	0	0	0	0
Gly-Lys	0	0	0	0	0	0	0	0	0	0	0
Gly-Met	4	2	4	0	2	0	0	0	0	0	0
Gly-Phe	5	4	0	0	4	0	0	0	2	0	0
Gly-Phe-Phe	4	4	0	4	4	4	2	0	4	2	0
Gly-Pro	0	0	0	0	0	0	0	0	0	0	0
Gly-Ser	5	0	5	0	0	0	0	0	0	0	0
Gly-Thr	5	1	5	0	1	0	0	0	0	0	0
Gly-Trp	5	4	0	2	4	0	0	0	4	0	0
Gly-Tyr	5	4	0	0	4	0	0	0	2	0	0
Gly-Val	5	4	0	0	4	0	0	0	0	0	0
His-Ala	4	2	0	4	2	2	2	2	4	2	0
His-Asp	5	3	0	0	4	0	0	0	1	0	0
His-Glu	4	4	0	0	4	0	0	0	0	0	0
His-Gly	5	4	0	0	4	0	0	0	4	0	0
His-His	0	0	0	0	0	0	0	0	0	0	0
His-Leu	5	4	0	3	4	2	2	3	4	2	0
His-Lys	0	0	0	0	0	0	0	0	0	0	0
His-Met	5	4	0	3	4	4	3	4	4	4	0
His-Pro	4	2	2	2	2	2	2	2	3	2	1
His-Ser	5	4	0	2	4	3	3	2	4	2	0
His-Trp	4	2	0	2	2	2	2	2	1	2	0
His-Tyr	5	4	0	4	4	3	3	4	4	3	0

	59A (wt)	opt1Δ opt2Δ Δdal5	fot1fot2Δ	PepTrKO ::*FOT1*	PepTrKO ::*FOT1-FOT2*	PepTrKO ::*FOT2*	PepTrKO ::*FOT2Tm*	PepTrKO ::*FOT3*	PepTrKO ::*FOTX*	PepTrKO ::*FOTY*	PepTrKO
His-Val	5	4	0	3	3	1	1	3	4	2	0
Ile-Ala	5	5	0	4	4	4	4	4	4	4	0
Ile-Arg	5	5	5	4	4	4	4	4	4	4	0
Ile-Asn	4	4	0	4	4	4	4	4	4	4	0
Ile-Gln	5	5	0	4	4	4	4	4	4	4	0
Ile-Gly	5	4	0	0	4	0	0	0	1	0	0
Ile-His	5	4	0	2	4	1	2	2	4	0	0
Ile-Ile	5	4	0	2	4	3	3	3	4	2	0
Ile-Leu	4	4	0	4	4	4	4	2	4	2	0
Ile-Met	5	4	0	3	4	3	4	3	4	3	0
Ile-Phe	5	3	0	2	4	3	3	2	4	3	0
Ile-Pro	1	0	0	0	0	0	0	0	0	0	0
Ile-Ser	5	5	0	2	4	2	4	2	4	2	0
Ile-Trp	4	2	0	2	2	2	2	2	4	2	0
Ile-Tyr	4	2	0	2	2	3	2	2	4	3	0
Ile-Val	5	4	0	4	4	4	4	4	4	4	0
L-Glutamine	4	4	4	4	4	4	4	4	4	4	4
Leu-Ala	5	5	0	4	5	4	4	4	4	4	0
Leu-Arg	5	5	5	4	5	4	4	5	5	4	0
Leu-Asn	5	4	0	4	4	4	3	2	4	1	0
Leu-Asp	4	3	0	0	3	0	0	0	0	0	0
Leu-β-Ala	1	2	0	0	2	0	0	0	0	0	0
Leu-D-Leu	0	0	0	0	0	0	0	0	0	0	0
Leu-Glu	5	3	0	0	3	0	0	0	0	0	0
Leu-Gly	5	3	0	0	4	0	0	0	0	0	0
Leu-Gly-Gly	4	4	0	0	4	0	0	0	2	0	0
Leu-His	4	4	0	4	4	3	2	3	4	2	0
Leu-Ile	5	4	0	3	4	4	4	3	4	4	0
Leu-Leu	5	4	0	2	4	3	3	2	4	2	0
Leu-Leu-Leu	4	4	4	4	4	2	2	4	4	3	0
Leu-Met	5	5	0	3	4	4	4	2	4	4	0
Leu-Phe	5	4	0	3	4	4	4	2	4	4	0
Leu-Pro	0	0	0	0	0	0	0	0	0	0	0
Leu-Ser	4	4	0	2	4	2	2	0	4	0	0
Leu-Trp	3	3	0	4	3	3	2	2	3	1	0
Leu-Tyr	4	4	0	4	4	4	3	3	4	2	0
Leu-Val	4	4	0	4	4	4	4	3	4	2	0
Lys-Ala	0	0	0	0	0	0	0	0	0	0	0
Lys-Arg	4	4	0	4	4	4	3	2	4	1	0
Lys-Asp	0	0	0	0	0	0	0	0	0	0	0

	59A (wt)	opt1Δ opt2Δ Δdal5	fot1fot2Δ	PepTrKO ::*FOT1*	PepTrKO ::*FOT1-FOT2*	PepTrKO ::*FOT2*	PepTrKO ::*FOT2Tm*	PepTrKO ::*FOT3*	PepTrKO ::*FOTX*	PepTrKO ::*FOTY*	PepTrKO
Lys-Glu	0	0	0	0	0	0	0	0	0	0	0
Lys-Gly	0	0	0	0	0	0	0	0	0	0	0
Lys-Ile	0	0	0	0	0	0	0	0	0	0	0
Lys-Leu	0	0	0	0	0	0	0	0	0	0	0
Lys-Lys	0	0	0	0	0	0	0	0	0	0	0
Lys-Met	0	0	0	0	0	0	0	0	0	0	0
Lys-Phe	1	1	0	0	1	0	0	0	0	0	0
Lys-Pro	0	0	0	0	0	0	0	0	0	0	0
Lys-Ser	0	0	0	0	0	0	0	0	0	0	0
Lys-Thr	0	0	0	0	0	0	0	0	0	0	0
Lys-Trp	1	0	0	0	1	0	0	0	0	0	0
Lys-Tyr	0	0	0	0	0	0	0	0	0	0	0
Lys-Val	0	0	0	0	0	0	0	0	0	0	0
Met-Ala	2	2	0	4	2	4	3	4	4	4	0
Met-Arg	4	4	3	4	4	4	4	4	4	0	0
Met-Asp	4	4	0	1	4	2	2	0	4	0	0
Met-β-Ala	2	2	0	0	2	1	0	0	2	0	0
Met-Gln	4	4	3	4	4	3	4	4	4	4	0
Met-Glu	4	4	0	0	4	0	0	0	2	0	0
Met-Gly	3	4	0	0	4	0	0	0	2	0	0
Met-His	4	5	0	4	5	4	4	4	4	4	0
Met-Ile	4	4	0	4	4	4	4	4	4	4	0
Met-Leu	4	4	2	4	4	4	4	3	4	3	0
Met-Lys	0	0	0	0	0	0	0	0	0	0	0
Met-Met	4	4	0	4	4	4	4	4	4	4	0
Met-Phe	4	4	0	4	4	4	3	4	4	3	0
Met-Pro	3	4	0	0	3	0	0	0	2	1	0
Met-Thr	4	4	0	4	4	4	3	3	4	3	0
Met-Trp	3	3	0	4	3	4	3	2	3	2	0
Met-Tyr	4	4	0	4	4	4	3	4	4	2	0
Met-Val	4	4	0	4	4	4	4	4	4	4	0
Negative control	0	0	0	0	0	0	0	0	0	0	0
Phe-Ala	4	4	0	4	4	4	4	4	4	4	0
Phe-Asp	4	4	0	2	4	0	0	0	4	0	0
Phe-β-Ala	1	1	0	0	2	0	0	0	0	0	0
Phe-Glu	4	4	0	2	4	0	0	0	4	0	0
Phe-Gly	3	4	0	2	3	0	0	0	3	0	0
Phe-Gly-Gly	4	4	0	4	4	2	2	2	4	0	0
Phe-Ile	3	4	0	4	4	4	3	3	3	2	0
Phe-Met	4	4	0	4	4	4	3	4	4	3	0

	59A (wt)	opt1Δ opt2Δ Δdal5	fot1fot2Δ	PepTrKO ::*FOT1*	PepTrKO ::*FOT1-FOT2*	PepTrKO ::*FOT2*	PepTrKO ::*FOT2Tm*	PepTrKO ::*FOT3*	PepTrKO ::*FOTX*	PepTrKO ::*FOTY*	PepTrKO
Phe-Phe	4	4	0	4	4	4	2	2	3	2	0
Phe-Pro	3	3	0	0	3	0	0	0	0	0	0
Phe-Ser	4	4	0	4	4	4	2	4	4	4	0
Phe-Trp	2	2	0	2	2	2	0	1	2	0	0
Phe-Tyr	4	4	0	4	4	4	3	4	4	2	0
Phe-Val	4	4	0	4	4	4	3	3	4	2	0
Pro-Ala	0	0	0	0	0	0	0	0	0	0	0
Pro-Arg	4	4	2	2	4	0	0	1	4	0	0
Pro-Asn	0	0	0	0	0	0	0	0	0	0	0
Pro-Asp	0	0	0	0	0	0	0	0	0	0	0
Pro-Gln	2	0	1	0	0	0	0	0	0	0	0
Pro-Glu	0	0	0	0	0	0	0	0	0	0	0
Pro-Gly	0	0	0	0	0	0	0	0	0	0	0
Pro-Hyp	0	0	0	0	0	0	0	0	0	0	0
Pro-Ile	0	0	0	0	0	0	0	0	0	0	0
Pro-Leu	0	0	0	0	0	0	0	0	0	0	0
Pro-Lys	0	0	0	0	0	0	0	0	0	0	0
Pro-Phe	0	0	0	0	0	0	0	0	0	0	0
Pro-Pro	0	0	0	0	0	0	0	0	0	0	0
Pro-Ser	0	0	0	0	0	0	0	0	0	0	0
Pro-Trp	2	2	0	0	2	0	0	0	0	0	0
Pro-Tyr	0	0	0	0	0	0	0	0	0	0	0
Pro-Val	0	0	0	0	0	0	0	0	0	0	0
Ser-Ala	4	4	3	4	4	4	3	3	4	0	0
Ser-Asn	5	4	4	2	4	0	0	1	4	0	0
Ser-Asp	0	0	0	0	0	0	0	0	0	0	0
Ser-Gln	5	4	4	3	4	0	0	1	4	0	0
Ser-Glu	0	0	0	0	0	0	0	0	0	0	0
Ser-Gly	2	2	0	0	2	0	0	0	0	0	0
Ser-His	4	4	2	4	4	4	4	2	4	3	0
Ser-Leu	4	4	4	3	4	0	0	0	4	0	0
Ser-Met	4	4	3	4	4	4	4	2	4	4	0
Ser-Phe	4	4	0	4	4	4	3	0	4	2	0
Ser-Pro	3	3	0	0	3	0	0	0	0	0	0
Ser-Ser	4	4	0	0	3	0	0	0	1	0	0
Ser-Tyr	4	4	0	4	4	4	3	2	4	4	0
Ser-Val	3	4	0	3	4	4	2	2	4	2	0
Thr-Ala	2	4	0	3	4	4	3	2	4	2	0
Thr-Arg	3	4	2	4	4	4	3	2	4	3	0
Thr-Asp	0	0	0	0	0	0	0	0	0	0	0

	59A (wt)	opt1Δ opt2Δ Δdal5	fot1fot2Δ	PepTrKO ::*FOT1*	PepTrKO ::*FOT1-FOT2*	PepTrKO ::*FOT2*	PepTrKO ::*FOT2Tm*	PepTrKO ::*FOT3*	PepTrKO ::*FOTX*	PepTrKO ::*FOTY*	PepTrKO
Thr-Gln	5	4	0	4	4	2	1	2	4	0	0
Thr-Glu	4	3	0	0	3	0	0	0	2	0	0
Thr-Gly	2	3	0	0	3	0	0	0	0	0	0
Thr-Leu	4	4	0	4	4	3	2	0	4	0	0
Thr-Met	3	3	0	4	3	4	3	2	4	3	0
Thr-Phe	4	4	0	4	4	4	4	4	4	4	0
Thr-Pro	2	2	0	0	2	0	0	0	2	0	0
Thr-Ser	5	4	4	4	4	4	3	3	4	2	2
Trp-Ala	3	3	0	2	3	3	2	2	4	2	0
Trp-Arg	3	4	2	4	4	4	2	1	3	0	0
Trp-Asp	1	1	0	0	1	0	0	0	0	0	0
Trp-Glu	1	0	0	0	0	0	0	0	0	0	0
Trp-Gly	0	0	0	0	0	0	0	0	0	0	0
Trp-Leu	2	3	0	0	2	0	0	0	2	0	0
Trp-Lys	2	2	0	0	2	1	1	0	0	1	0
Trp-Phe	3	2	0	2	3	0	0	0	2	0	0
Trp-Ser	2	2	0	0	2	0	0	0	0	0	0
Trp-Trp	1	1	0	0	1	0	0	0	0	0	0
Trp-Tyr	3	3	0	4	3	4	3	0	4	2	0
Trp-Val	4	3	0	0	4	0	0	0	3	0	0
Tyr-Ala	4	4	0	4	4	4	4	4	4	4	0
Tyr-Gln	4	4	2	4	4	4	4	4	4	4	0
Tyr-Glu	2	3	0	1	3	0	0	0	3	0	0
Tyr-Gly	2	3	0	0	3	0	0	0	1	0	0
Tyr-Gly-Gly	4	4	0	1	4	0	0	0	4	0	0
Tyr-His	4	4	0	4	4	4	3	3	4	4	0
Tyr-Ile	4	4	0	4	4	4	3	3	4	3	0
Tyr-Leu	2	3	0	4	3	4	3	2	4	2	0
Tyr-Lys	0	1	0	0	0	0	0	0	0	0	0
Tyr-Phe	2	4	0	3	2	4	2	2	4	2	0
Tyr-Trp	3	3	0	4	3	4	3	4	4	2	0
Tyr-Tyr	4	4	0	4	4	4	4	4	4	4	0
Tyr-Val	4	4	0	4	4	4	3	3	4	2	0
Val-Ala	5	4	0	4	4	4	4	4	4	2	0
Val-Arg	4	4	2	4	4	4	4	4	4	4	0
Val-Asn	4	4	0	4	4	4	4	4	4	3	0
Val-Asp	4	4	0	0	4	0	0	0	1	0	0
Val-Gln	5	4	0	4	4	3	2	2	4	1	0
Val-Glu	2	2	0	0	2	0	0	0	0	0	0
Val-Gly	4	4	0	0	4	0	0	0	1	0	0

	59A (wt)	opt1Δ opt2Δ Δdal5	fot1fot2Δ	PepTrKO ::*FOT1*	PepTrKO ::*FOT1-FOT2*	PepTrKO ::*FOT2*	PepTrKO ::*FOT2Tm*	PepTrKO ::*FOT3*	PepTrKO ::*FOTX*	PepTrKO ::*FOTY*	PepTrKO
Val-His	4	4	0	4	4	3	4	4	3	4	0
Val-Ile	4	4	0	4	4	4	4	3	4	4	0
Val-Leu	4	4	0	4	4	4	3	2	4	2	0
Val-Lys	0	0	0	2	0	0	0	0	0	0	0
Val-Met	4	4	0	4	4	4	3	4	4	3	0
Val-Phe	4	4	0	4	4	4	4	4	4	3	0
Val-Pro	4	4	0	0	4	0	0	0	1	0	0
Val-Ser	5	4	0	3	4	1	0	0	4	0	0
Val-Tyr	2	4	0	4	3	4	3	2	4	2	0
Val-Tyr-Val	4	3	0	4	4	4	2	3	2	2	0
Val-Val	4	4	0	4	4	4	3	3	4	2	0

Table S4. Amino acid composition of N- and C-termini of oligopeptides consumed by Fot. Percentages are calculated according to the oligopeptide fraction transported by each single Fot (level consumption ≥ 1). Amino acids are categorized by type: hydrophobic (Ala, Val, Leu, Ile, Met, Phe, Trp, Gly, Pro and β-Ala), polar (Tyr, Ser, Thr, Gln, Asn), basic (His, Arg, Lys) and acidic (Glu, Asp, γ-Glu) amino acids.

	**Fot1Fot2**		**Fot1**		**Fot2**		**Fot2Tm**		**Fot3**		**FotX**		**FotY**	
Terminus/ Amino acid	N	C	N	C	N	C	N	C	N	C	N	C	N	C
Ala	10.47	7.33	11.45	9.16	11.97	10.26	12.93	10.34	11.82	10.91	10.53	7.89	9.09	10.10
Val	8.90	6.81	10.69	7.63	11.11	8.55	10.34	8.62	10.91	9.09	10.53	7.89	12.12	10.10
Leu	9.42	6.81	9.92	7.63	11.11	7.69	11.21	7.76	10.91	7.27	9.21	7.24	12.12	7.07
Ile	7.33	4.19	9.92	5.34	11.11	5.98	11.21	6.03	11.82	6.36	9.21	4.61	12.12	7.07
Met	8.90	5.24	9.92	6.87	11.97	7.69	11.21	7.76	10.91	8.18	11.18	5.92	12.12	9.09
Phe	7.33	7.85	9.16	8.40	7.69	8.55	6.90	8.62	8.18	6.36	7.89	7.89	7.07	10.10
Trp	5.24	7.33	3.05	6.87	3.42	5.98	3.45	5.17	1.82	5.45	3.95	6.58	3.03	5.05
Gly	7.85	7.85	3.05	3.05	2.56	1.71	2.59	1.72	1.82	1.82	3.95	6.58	3.03	0.00
Pro	1.05	3.66	0.76	0.76	0.00	0.85	0.00	0.86	0.91	0.91	0.66	2.63	0.00	2.02
β-Ala	0.00	1.57	0.00	0.00	0.00	0.85	0.00	0.00	0.00	0.00	0.00	0.66	0.00	0.00
Tyr	6.28	7.33	8.40	9.16	7.69	9.40	7.76	9.48	8.18	9.09	7.89	8.55	9.09	11.11
Ser	6.28	4.71	6.87	4.58	5.13	5.13	5.17	4.31	6.36	3.64	6.58	5.26	5.05	4.04
Thr	5.24	1.57	5.34	1.53	5.98	1.71	6.03	1.72	5.45	1.82	5.92	1.32	5.05	1.01
Gln	1.57	4.71	0.76	6.11	0.00	5.13	0.86	6.03	0.00	6.36	0.66	5.26	0.00	4.04
Asn	1.05	2.62	0.76	3.82	0.85	2.56	0.86	3.45	0.91	4.55	0.66	3.29	1.01	3.03
His	5.76	4.19	6.11	6.11	6.84	6.84	6.90	6.90	7.27	7.27	6.58	5.26	8.08	7.07
Arg	2.09	5.76	0.76	8.40	0.85	8.55	0.86	8.62	0.91	10.00	1.32	7.24	0.00	7.07
Lys	1.57	1.57	0.76	1.53	0.85	1.71	0.86	1.72	0.91	0.91	0.66	0.66	1.01	2.02
Glu	2.09	5.24	1.53	1.53	0.00	0.00	0.00	0.00	0.00	0.00	1.97	2.63	0.00	0.00
Asp	1.05	3.66	0.00	1.53	0.00	0.85	0.00	0.86	0.00	0.00	0.00	2.63	0.00	0.00
γ-Glu	0.52	0.00	0.76	0.00	0.85	0.00	0.86	0.00	0.91	0.00	0.66	0.00	0.00	0.00

Table S5. Binding motifs for transcription factors (TFs) found in FOT promoter regions. An upstream region of 500 bp was analyzed to find transcription motifs with >95% of maximum score in expert-curated, non-dubious YeTFaSCo database.

TF	Binding sequence	Promoter	Position (strand)	Function
ASH1	CTGAT	*FOTY*	76-80 (-)	Repressor that negatively regulates mating type switching and G1/S transition of mitotic cells.
BAS1	GAGTCA	*FOTY*<br>*FOTY*	173-178 (+)<br>357-362 (+)	Regulation of basal and induced expression of genes of the purine and histidine biosynthesis pathways as well as genes involved in regulation of meiotic recombination.
CUP9	AATGTGTCAA	*FOTX, FOT1, FOT3*<br>*FOTY*<br>*FOT2Tm, FOT2*<br>*FOTY*	183-192 (-)<br>184-193 (-)<br>191-200 (-)<br>444-453 (-)	Represses transcription of the oligopeptide transporters *PTR2* and *OPT2*, while upregulating the allantoate permease *DAL5*.
DAL80	CGCGATAG	*FOT2Tm, FOT2*	306-313 (+)	Negative regulator of genes in multiple nitrogen degradation pathways.
DAT1	TTTTTTTTTTT	*FOTX, FOT1*	214-224 (+)	Relocalizes to the cytosol in response to hypoxia.
GLN3	GATAA	*FOTY*<br>*FOTY*<br>*FOT2Tm, FOT2*<br>*FOTX, FOT1*<br>*FOT3*<br>*FOT3*<br>*FOTX, FOT1*<br>*FOTX, FOT1*<br>*FOT3*<br>*FOT2Tm, FOT2*<br>*FOTX, FOT1*<br>*FOTX, FOT1, FOT3*	161-165 (-)<br>209-213 (-)<br>216-220 (-)<br>245-249 (-)<br>287-291 (-)<br>318-322 (+)<br>329-333 (-)<br>360-364 (+)<br>429-433 (+)<br>45-49 (-)<br>471-475 (+)<br>7-11 (+)	Activator of genes regulated by the nitrogen catabolite repression system.
GTS1	TACCAA	*FOT3*<br>*FOTX, FOT1*	297-302 (+)<br>339-344 (+)	Involved in endocytosis and regulation of phosphatidylinositol biosynthesis.
HAA1	GCGGGG	*FOTY*<br>*FOT2Tm, FOT2*	290-295 (-)<br>332-337 (-)	Activator of genes involved in adaptation to weak acid stress.
HMRA2	CATGTAAA	*FOTY*<br>*FOT2Tm, FOT2*	348-355 (-)<br>370-377 (-)	Required along with a1p for inhibiting expression of the HO endonuclease in a/alpha HO/HO diploid cells.
HSF1	TTCTAGAA<br>ATGGAACG	*FOT2Tm, FOT2*<br>*FOTX, FOT1, FOT3*	274-281 (2; +, -)<br>32-39 (+)	Involved in sensibility to rapamycin.

TF	Binding sequence	Promoter	Position (strand)	Function
MATALPHA2	TCATGT	*FOT2Tm*, *FOT2*	174-179 (+)	Represses specific genes in haploids.
		FOT3	276-281 (+)	
		FOTX, *FOT1*	318-323 (+)	
		FOTY	351-356 (-)	
		FOT2Tm, *FOT2*	373-378 (-)	
MOT2	ATATA	*FOT2Tm*, *FOT2*	105-109 (-)	Involved in protein ubiquitination and catabolism, mRNA decapping, and regulation of transcription elongation.
		FOTX, *FOT1*, *FOT3*	134-138 (-)	
		FOTX, *FOT1*, *FOT3*	171-175 (-)	
		FOTX, *FOT1*, *FOT3*	172-176 (+)	
		FOT2Tm, *FOT2*	202-206 (-)	
		FOTY	232-236 (+)	
		FOTY	29-33 (+)	
		FOT2Tm, *FOT2*	492-496 (+)	
		FOTY	58-62 (+)	
		FOTX, *FOT1*, *FOT3*	99-103 (-)	
MSN1	TATGTCCT	*FOTY*	85-92 (+)	Activator of stress-responsive genes.
MSN4	CCCCT	*FOT3*	368-372 (+)	Activator of stress-responsive genes.
		FOTX, *FOT1*	410-414 (+)	
PUT3	CCCCGGGAGA	*FOT2Tm*, *FOT2*	332-341 (+)	Regulates proline utilization genes.
RGM1	CCCCT	*FOT3*	368-372 (+)	Regulation of genes involved in monosaccharide catabolism and aldehyde metabolism.
		FOTX, *FOT1*	410-414 (+)	
RGT1	CGGAAAAATT	*FOTX*, *FOT1*, *FOT3*	54-63 (+)	Regulation of glucose transporter genes in response to glucose.
RSC3	CGCGCGG	*FOT2Tm*, *FOT2*	303-309 (-)	Involved in nucleosome positioning, transcription regulation and ploidy maintenance.
SUT1	CGCGGGG	*FOTY*	290-296 (-)	Positively regulates sterol uptake genes under anaerobic conditions; involved in hypoxic gene expression.
TOS8	TTTGACAG	*FOTY*	22-29 (-)	Paralog of *CUP9*; induced during meiosis and under cell-damaging conditions.
		FOTX, *FOT1*	259-266 (+)	
		FOTY	384-391 (+)	
		FOT3	461-468 (+)	
UPC2	TCGTATA	*FOTX*, *FOT1*, *FOT3*	173-179 (-)	Induces sterol biosynthetic genes upon sterol depletion.
USV1	AGGGG	*FOT3*	368-372 (-)	Regulation of genes involved in growth on non-fermentable carbon sources, response to salt stress and cell wall biosynthesis.
		FOTX, *FOT1*	410-414 (-)	
YAP3	TTACTAA	*FOT3*	492-498 (-)	Involved in resistance to rapamycin, acid and DNA-damaging agents, among others.
